# Discovery and molecular mechanism of potent neutralizing antibody from humanized mice with respiratory syncytial virus

**DOI:** 10.1371/journal.ppat.1013674

**Published:** 2025-11-17

**Authors:** Zheng Zhang, Rui Feng, Long Zhang, Qi Yang, Xuehua Chen, Xiaoxiao Wang, Cui Nie, Wei Peng, Xiangxi Wang, Ling Zhu, Yu Guo, Zixian Sun

**Affiliations:** 1 Graduate School of Guangzhou Medical University, Panyu District, Guangzhou, China; 2 Guangzhou National Laboratory, Guangzhou International Bio Island, Guangzhou, Guangdong Province, China; 3 State Key Laboratory of Medicinal Chemical Biology and College of Life Sciences, Nankai University, Tianjin, P. R. China,; 4 CAS Key Laboratory of Infection and Immunity, National Laboratory of Macromolecules, Institute of Biophysics, Chinese Academy of Sciences, Beijing, China; 5 Harbour Biomed (Suzhou) Co. Ltd., Suzhou, China; University of Texas Medical Branch / Galveston National Laboratory, UNITED STATES OF AMERICA

## Abstract

Respiratory syncytial virus (RSV) is a leading cause of lower respiratory tract infections among infants and older adults, posing a significant threat to global public health. The prophylactic use of neutralizing antibodies (nAbs) underscores the need to understand elite RSV antibody neutralization mechanisms, which is fundamental for developing next-generation therapies with enhanced potency and broader activity. In this study, we utilized H2L2 transgenic mice encoding human immunoglobulin variable regions for immunization and successfully screened multiple antibodies with significant neutralizing activity using the Beacon Optofluidic system. One of these antibodies, PR306007, exhibited significantly superior broad-spectrum neutralization against both RSV-A and B subgroups. Cryo-electron microscopy (Cryo-EM) structural analysis revealed that PR306007 binds to a unique epitope that overlaps with antigenic sites II and V of the F protein, with its primary binding regions located at the base of the α6 and α7 helices of site II, and residues S173 and N175 of site V. This binding mode offers valuable insights into enhanced neutralization activity and potentially reduces the risk of emerging immune evasive mutants. Furthermore, PR306007 showed potent *in vivo* antiviral activity against RSV infection and demonstrated good efficacy against both lower and upper respiratory tract infections, making it a promising prophylactic candidate for broad prevention. These findings provide new insights for the future development of RSV vaccines or nAbs.

## Author summary

Respiratory syncytial virus (RSV) poses a significant burden on global public health, presenting a serious health threat to infants, young children, the elderly, and other vulnerable populations, including individuals with immunodeficiencies. The ongoing development of neutralizing antibodies against RSV not only holds substantial scientific value but also promises widespread clinical applications. In this study, we reported for the first time the isolation of the neutralizing antibodies against RSV from immunized humanized mice. The neutralizing antibody PR306007 exhibits potent activity against both RSV-A and B subgroups. We elucidated the cryo-electron microscopy structure of PR306007, a potent activity and broad-spectrum neutralizing antibody, in complex with the RSV pre-F antigen. Through structural and sequence analyses, we revealed that PR306007 bound to a unique epitope across the antigenic sites II and V of the F protein. Notably, the heavy chain gene of PR306007 is encoded by IGHV1–8, which is relatively rare within the known repertoire of antiviral antibody lineages. Furthermore, PR306007 demonstrated effective prophylactic effect in animal experiments, underscoring its potential as a broad-spectrum therapeutic against RSV. These findings offer valuable insights and guidance for the development of next-generation therapies and vaccines.

## Introduction

Since the discovery of respiratory syncytial virus (RSV) in humans in 1957, it has posed a significant threat to global health [1,2]. The World Health Organization (WHO) estimates that RSV-related lower respiratory tract infections (LRTIs) account for approximately 33 million cases annually, with incidence rates continuing to escalate [3]. The genome of RSV encodes two non-structural proteins and nine structural proteins, among which the F protein on the viral surface, due to its critical role in the viral entry process, has emerged as a major target for antibody and vaccine development [4]. The F protein, as Type-I membrane fusion protein, forms a trimeric structure as an F2-F1 heterodimer on the viral envelope and exists in two conformations: a metastable pre-fusion (pre-F) conformation and a stable post-fusion conformation [5]. Previous studies have revealed that, despite the pre-F and post-fusion (post-F) forms of the RSV fusion protein sharing roughly 50% of their antigenic epitopes, the neutralizing antibodies (nAbs) elicited by the pre-F protein exhibit a markedly higher affinity and efficacy. Additionally, there is a significantly greater activation of memory B cells in response to the pre-F protein as compared to the post-F protein [6]. The structural engineering efforts have successfully stabilized the F protein in its pre-F conformation, which has been designated as DS-Cav1 [5,7]. To date, the pre-F protein as a key antigenic target for the development of RSV vaccines and therapeutic interventions.

Monoclonal antibody (mAb) therapeutics targeting the RSV F protein represent a well-established and effective strategy for both prophylaxis and treatment, particularly for individuals who are immunocompromised, or regarding vaccine enhanced respiratory disease (ERD) [8]. Currently, several monoclonal antibodies targeting RSV are undergoing clinical trials, among which palivizumab and nirsevimab have received approval for the prevention of RSV infections in infants. However, palivizumab is associated with issues such as frequent administration and high costs, and it is limited use in high-risk infants and young children [9–11]. Similarly, nirsevimab raises potential concerns regarding immune escape [4,12,13]. Therefore, deep exploration of the molecular mechanism of neutralizing antibodies (nAbs) would significantly advance the development of next-generation therapies.

At least six antigenic sites have been reported on the RSV F glycoprotein: Ø, V, II, III, IV, and I. The Ø and V sites are exclusively associated with the pre-F conformation, whereas sites I, II, III, and IV are observed in both pre-F and post-F conformations, although site I predominantly elicits post-F specific antibodies [14,15]. Notably, the site II is the binding site for the commercial antibody palivizumab, which is highly conserved and located within the helix-loop-helix structure of RSV F residues 254–277 [16]. Studies demonstrate that the sites Ø and V in the pre-F conformation can induce strong neutralizing antibody responses [4,17]. The site Ø exhibits high variability among RSV subtypes and extensive glycosylation, prompting a shift in research focus toward the site V. The site V, located between the sites Ø and III, contains structural elements from α3-α4 and β3-β4 [18]. Currently, the development of neutralizing antibodies against RSV primarily involves isolating antibodies from diseased or recovered individuals or immunized mice. However, murine antibodies are unable to effectively trigger complement and Fc receptor effector functions, limiting their clinical application [19]. Moreover, the human-derived antibodies originate from a limited number of dominant germline families and recognize similar epitopes [20].

Harbour H2L2 transgenic mice possess complete human variable regions of both heavy and light chains, allowing for the isolation of antibodies without additional humanization murine mAbs, thus simplifying the modification process and significantly reducing the risk of development failure. Furthermore, owing to the disparity in the humoral immune response between mice and humans, it is possible for novel epitopes distinct from those previous in humans to emerge [21]. And this approach requires less time and provide greater control compared to the isolation of antibodies from recovered individuals [22].

In this study, we utilized the H2L2 transgenic mouse platform and advanced antibody screening technology to identify RSV-specific monoclonal antibodies with low immunogenicity, high affinity, and low toxicity. Immunization with RSV pre-F protein yielded 331 paired humanized antibody sequences. Among them, six antibodies demonstrated strong neutralizing activity against RSV A2 in vitro, with NT_50_ values below 20 μg/mL. Notably, PR306007 exhibited superior neutralization activity both RSV A and B subtypes, and showed good affinity. Using single-particle cryo-electron microscopy (cryo-EM) and three-dimensional reconstruction techniques, we determined the structure of the RSV pre-F and PR306007 fragment antigen-binding (Fab) complex. The analysis revealed that PR306007 interacts with residues at both sites II and V, binding to sub-sites IIa and IIb within site II. Furthermore, the heavy chain gene of PR306007 is encoded by IGHV1–8 that is relatively rare within the known repertoire of antiviral antibody lineages. We further assessed the *in vivo* efficacy of PR306007 in an animal model, confirming its sustained strong neutralizing activity and preventive effects. Our findings indicate that PR306007 has the potential for development as a broad-spectrum, highly effective neutralizing antibody, providing new insights into RSV conserved epitopes. These discoveries offer valuable perspectives for antibody modification and therapeutic development, guiding preventive strategies and vaccine design against RSV infection.

## Results

### Isolation of antibodies from humanized mouse

**Fig 1:**
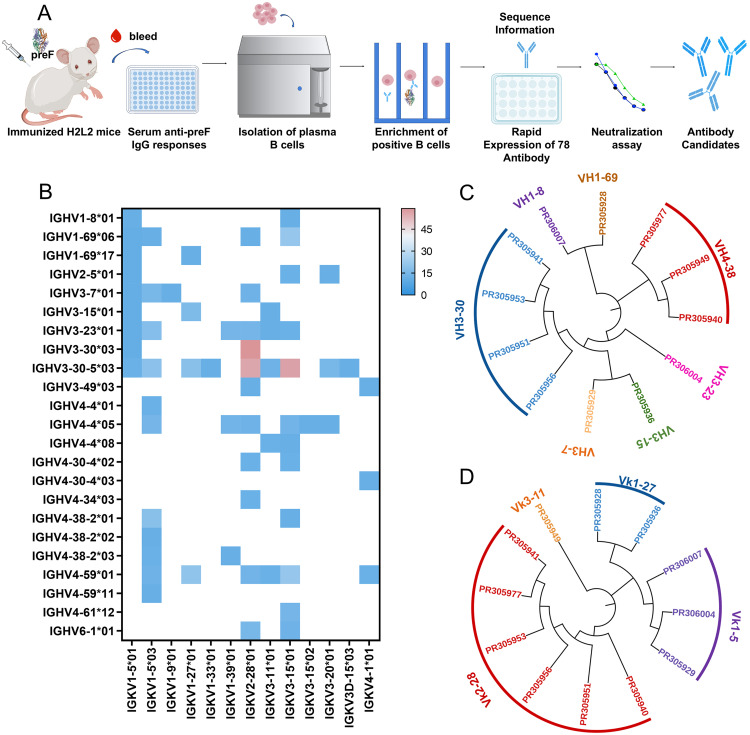
Antibodies isolated from H2L2 transgenic mice by single-cell sequencing. (A) Schematic diagram of experimental design of antibody discovery. Harbour H2L2 transgenic mice were immunized with Respiratory syncytial virus (RSV) pre-fusion (pre-F) protein, and the serum titers of antigen-specific antibodies in H2L2 mice were determined using enzyme-linked immunosorbent assay (ELISA). Spleen and bone marrow cells were harvested from these mice to isolate plasma B cells. Beads conjugated with RSV pre-F protein were employed to enrich B cells that specifically bind to RSV pre-F. Single B cell sequencing was conducted to recover the paired heavy- and light-chain sequences from each cell. Based on the sequence information, 78 sequences were selected for rapid construction and expression. The resulting antibodies were screened using neutralization assays of cell supernatants to identify candidate antibodies. (B) The chart shows the heavy- and light-chain germline gene pairing of 331 antibodies based on IMGT. The colors in the heatmap represent the number of antibodies identified with various coding genes, with the germline genes for the heavy and light chains displayed along the vertical and horizontal axes, respectively. The right of the graph indicates the abundance of the genes, with darker colors representing the higher abundance of the genes and lighter colors representing the lower abundance of the genes. All the data of this figure can be found in the S1 Table. (C, D) Phylogenetic analysis of the relationship between antibody variable gene segments and germline. The relationships between the heavy and light chain variable regions and the germlines from H2L2 transgenic mouse antibodies are shown. The analysis of the germline nucleic acid sequence of the antibodies was performed with IMGT/V-QUEST.

Previous research primarily isolated RSV antibodies from immunized mice, convalescent patients, or vaccine recipients. Here, considering the intricacies of humanization and the efficiency of selecting diverse antigenic epitopes, we immunized Harbour H2L2 humanized mice with RSV pre-F protein (DS-Cav1) (Fig 1A). After each immunization, serum samples were collected from the humanized mice, and enzyme-linked immunosorbent assay (ELISA) was employed to analyze serum binding to RSV pre-F (S1 Fig). Following multiple boost immunizations, we selected six mice with high serum titers and collected their spleen and bone marrow cells to isolate plasma B cells (S2 Fig).

We utilized the Beacon system’s OptoSelect chip to capture individual plasma B cells specific to RSV F protein. These cells were transferred to a 96-well plate pre-filled with lysis buffer, yielding a total of 384 positive single cells. The variable regions of heavy chains (VH) and light chains (VL) from these positive single cells were sequenced to extract naturally paired monoclonal antibody sequences, resulting in 331 VH-VL pairs. We performed gene sequence analysis of the isolated antibodies by using the International ImMunoGeneTics Information System (IMGT) database and IgBLAST (Fig 1B). The germline distribution was also diverse, with certain RSV F-specific repertoires skewed toward VH germline genes (VH1–69 and VH4-30-4). A large portion of antibodies utilized the IGHV3 germline genes, which is consistent with recent studies that a strong IGHV3 usage bias was found in the anti-virus antibody sequence repertoire in humans [23,24]. Based on sequence similarity, 78 antibody sequences were selected, and their phylogenetic relationships were illustrated (S3 Fig). To further isolate potent neutralizing antibodies, we synthesized these antibodies and transiently expressed them in HEK293 cells in a 24-well format, collecting the supernatant for viral neutralization assays (Fig 1A). From the neutralization results, we identified 12 antibodies with neutralization activity above 1.6 logs (S4 Fig). Further germline phylogenetic analysis revealed that the VH domains of these antibodies were encoded by three different progenitor lineages: VH1, VH3, and VH4, while their VL domains belonged to three distinct lineages: Vk1, Vk2, and Vk3 (Fig 1C and 1D). These lineages of antibodies similar to the preferred families of human antibodies repertoire [25].

### Characterization of a panel of potent neutralizing antibodies

**Fig 2:**
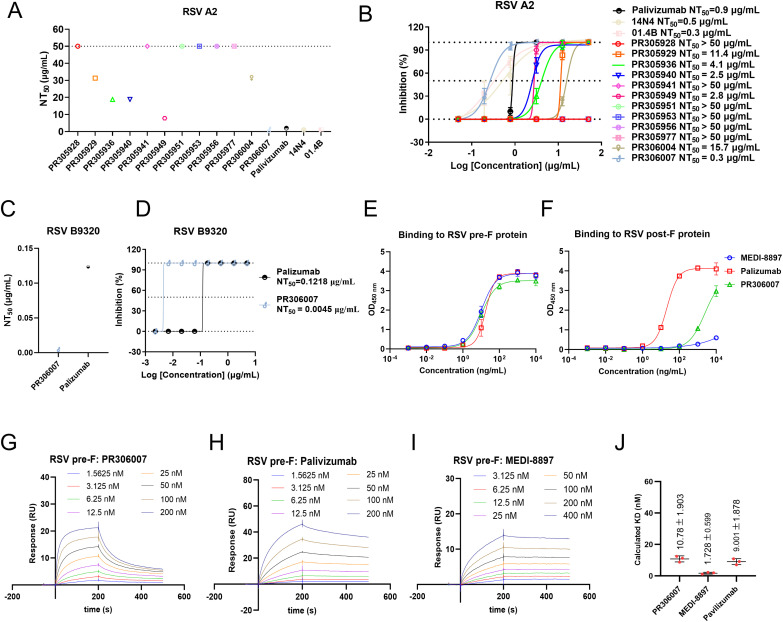
In vitro characterization of selected antibodies. Twelve monoclonal antibody strains were evaluated simultaneously by the cytopathic effect (CPE) assay (A) and focus-forming assay (FFA) (B) methods to neutralize live RSV A2 virus, and six of them showed antiviral activity, with PR306007 being more effective than palivizumab and 14N4 (positive controls). Data represents one of three independent experiments, shown as mean values ±standard deviation (S.D.). The data for Fig 2B can be found in S2 Table. (C, D) The neutralizing activity of PR306007 against the RSV B9320 virus was evaluated using the CPE method. Palivizumab (site II binder) was used as a positive control. Data were represented as the mean ± S.D. from n = 3 biologically independent experiments. The data for Fig 2D can be found in S3 Table. (E, F) The binding activity of PR306007 to the purified recombinant pre-F (DS-Cav1) and post-F from RSV A2 was measured using an enzyme-linked immunosorbent assay (ELISA). Data represents one of three independent experiments, displayed as the mean±S.D. of three technical replicates. Palivizumab and MEDI-8897 served as controls. (G, H, I) These antibodies (PR306007, MEDI-8897, and palivizumab) were tested for binding capability to the RSV pre-F (stain A2) by Biacore sensorgrams from triplicate experiments and representative results from one of these experiments are presented here. The original curves of three experiments are presented in S6 Fig. Palivizumab and MEDI-8897 served as controls. (J) These antibodies (PR306007, MEDI-8897, and palivizumab) were tested for their capability to bind the RSV pre-F by Biacore sensorgrams in triplicate experiments. The calculated KD values for the binding of each antibody to pre-F are summarized as the mean values of three experiments with standard deviation. The original curves of three experiments are presented in S6 Fig. Palivizumab and MEDI-8897 served as controls.

To better assess the neutralization potencies of these 12 nAbs, we synthesized their genes, expressed and purified them in the HEK293 expression system, and performed neutralization assays on authentic RSV A2 strain (S5 Fig). Palivizumab, 14N4 and 01.4B were used as positive controls. We employed cytopathic effect (CPE) assay and focus-forming assay (FFA) to determine the neutralizing activity of the 12 antibodies against the A2 strain. Six antibodies exhibited geometric mean half-maximal neutralizing titers (NT_50_) higher than 50 μg/mL, indicating poorly neutralizing activity (Fig 2A and 2B). However, another six antibodies could effectively neutralize A2 in vitro, showing NT_50_ values ranging from 0.3 to 15.7 μg/mL (Fig 2B). Notably, PR306007 had an NT_50_ value of 0.3 μg/mL, comparable to or even superior to positive controls (Fig 2A and 2B). Further neutralization testing against the authentic RSV B9320 strain also demonstrated that PR306007 exhibits remarkable neutralizing efficacy, with an NT_50_ value of 0.0045 μg/mL (Fig 2C and 2D). These findings indicated the broad neutralizing potential of PR306007 as a promising candidate for further antibody development.

Additionally, we conducted the ELISA assay to evaluate the binding affinity of PR306007 in comparison to palivizumab and MEDI-8897, targeting both pre- F and post-F conformations. The results indicated that PR306007 exhibits a pronounced preference for binding to the pre-F protein, while retaining comparatively weak binding to the post-F, with its EC_50_ for post-F binding exceeding 2000 ng/mL (Fig 2E and 2F). This suggests that the binding site of this antibody may reside in a region subject to conformational changes. Then, we analyzed the binding affinity of PR306007 to the RSV pre-F protein by surface plasmon resonance (SPR). The affinity results revealed that PR306007 binds to the RSV pre-F protein with the nanomolar affinity, comparable to MEDI-8897 and palivizumab, demonstrating the high binding activity of PR306007 (Figs 2G–2J and S6).

### Cryo-EM structure of the PR306007-RSV pre-F Complex

**Fig 3:**
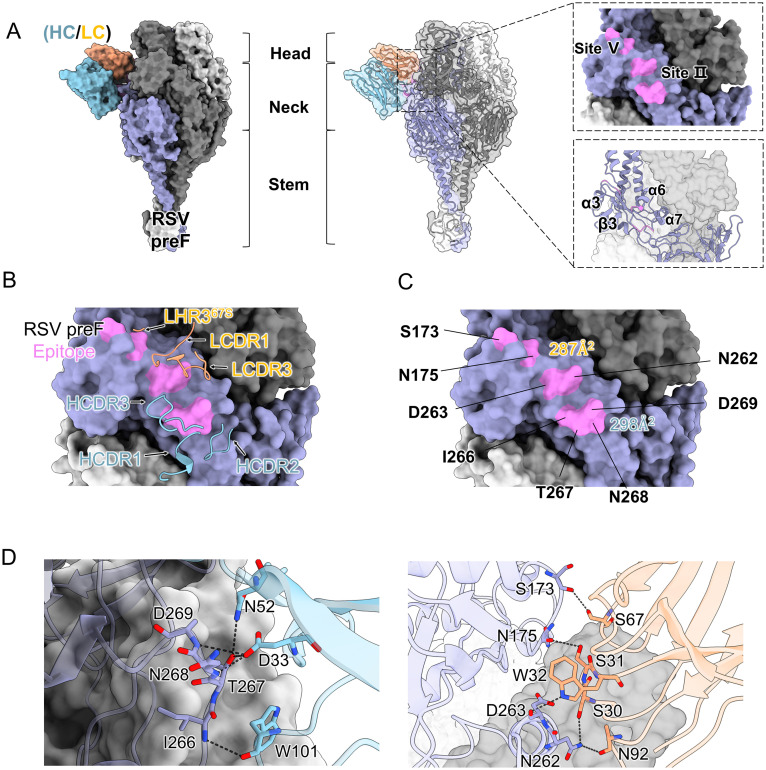
Structure of the RSV pre-F in complex with the neutralizing antibody (nAb) of PR306007. (A) Cryo-EM structure of the RSV pre-F ectodomain in complex with the nAb PR306007 fragment antigen-binding (Fab), as represented by the Cryo-EM map. Three RSV pre-F protomers are depicted in different colors, colored grey, dark grey, and purple. The variable regions of heavy chain (VH) is shown in blue, while the variable regions of light chain (VL) is shown in orange. In focus, only the variable regions of the Fab fragments are shown in the structure, while the constant regions are not modeled. The binding interface between RSV pre-F and PR306007 is framed and shown as a close-up view in the right-hand panel. This close-up view illustrates the epitope of PR306007 on pre-F, which interacts with residues at sites II and V of pre-F. (B) Interaction analysis between the RSV pre-F and PR306007. RSV pre-F are shown as molecular surfaces. PR306007-VH (blue): CDR1, CDR2, CDR3; PR306007-VL (orange): CDR1, CDR3, and FR-L3. (C) The binding region of PR306007 on the RSV pre-F. The binding region is colored magenta, and the buried surface areas (BSAs) of the light and heavy chains are 287 Å2(orange) and 298 Å2(blue). (D) Close-up view of RSV pre-F and PR306007 interaction interface. The variable domain of PR306007 and one pre-F protomer are shown as cartoon, colored blue (heavy chain), orange (light chain), and purple (pre-F), respectively. For clarity, the key critical contact residues are labeled and shown as sticks with oxygen atoms colored red and nitrogen atoms colored blue. Hydrogen bonds are depicted as black dotted lines.

To elucidate the neutralizing molecular mechanism of PR306007, we prepared the Fab and the stable pre-fusion glycoprotein F variant DS-Cav1 following previously established protocols [5] (S7 and S8 Figs). Subsequently, the purified Fab of PR306007 was incubated with RSV pre-F protein. Molecular exclusion chromatography analysis confirmed the formation of an immune complex between PR306007 and the A2 pre-F protein (S7 Fig). Using cryo-electron microscopy and single-particle three-dimensional reconstruction, we determined the structure of the RSV-F trimer complexed with PR306007 Fab at overall resolution of 4.08 Å (S9 and S10 Figs). The electron density map clearly showed the components of the pre-F protein and PR306007 Fab, with the pre-F trimer displaying a lollipop-like conformation, where three protomers closely surround a threefold symmetry axis, and each Fab interacts with an individual protomer (S9 Fig). This indicates that PR306007 prefers targets individual protomers rather than the entire trimer, which corresponds with the observed higher off-rate (Kd) compared to the other two antibodies (Figs 2G–2I and S6). Due to the flexibility and physiological nature of the viral-host infection, we next performed the local refinement of the complex structure of single Fab and protomer to improve the resolution of the antibody interface regions and better understand the molecular interactions (Figs 3A and S9-S11).

Upon accurately model building, we were able to precisely identify the epitope of PR306007 on the pre-F protein. The main interacting residues are located within the helix-turn-helix motif of site II and some residues of site V from one F protomer (Fig 3A, 3B and 3C). The epitope of PR306007 largely overlaps with the antigenic II site, which is conserved in both pre-F and post-F conformations [16]. The PR306007 heavy chain binds to RSV-F with 298 Å^2^ of surface area, and the light chain buries 287 Å^2^, resulting in a total buried surface area of 585 Å^2^ (Fig 3C). The heavy chain HCDR1, HCDR2 and HCDR3 as well as the light chain LCDR1, LCDR3 and LFR-3 of PR306007 interact with RSV F (Fig 3B). The four CDRs (HCDR1, HCDR2, HCDR3 and LCDR3) predominantly contact residues on antigenic site II, and the LCDR1 and LFR-3 contacts antigenic site V (Fig 3B).

The interactions between N52, W101, and D33 of the HCDRs of PR306007 and the bulge region constituted by T267, I266, N268, and D269 of pre-F establish a relatively stable and extensive hydrogen bond network. The interactions were further stabilized by electrostatic interactions among S67, S31, W32, and S30 of the LCDRs with S173, N175, D263, and N262 (Figs 3C, 3D, and S11). Structural analysis of the complex structure reveals that PR306007 simultaneously recognizes an epitope that spans two known antigenic sites, exhibiting strong interactions with the α6 and α7 helices of the RSV pre-F site II and the residues S173 and N175 of the site V (Fig 3C and 3D).

### Analysis of the neutralization mechanism of potent antibody PR306007

**Fig 4:**
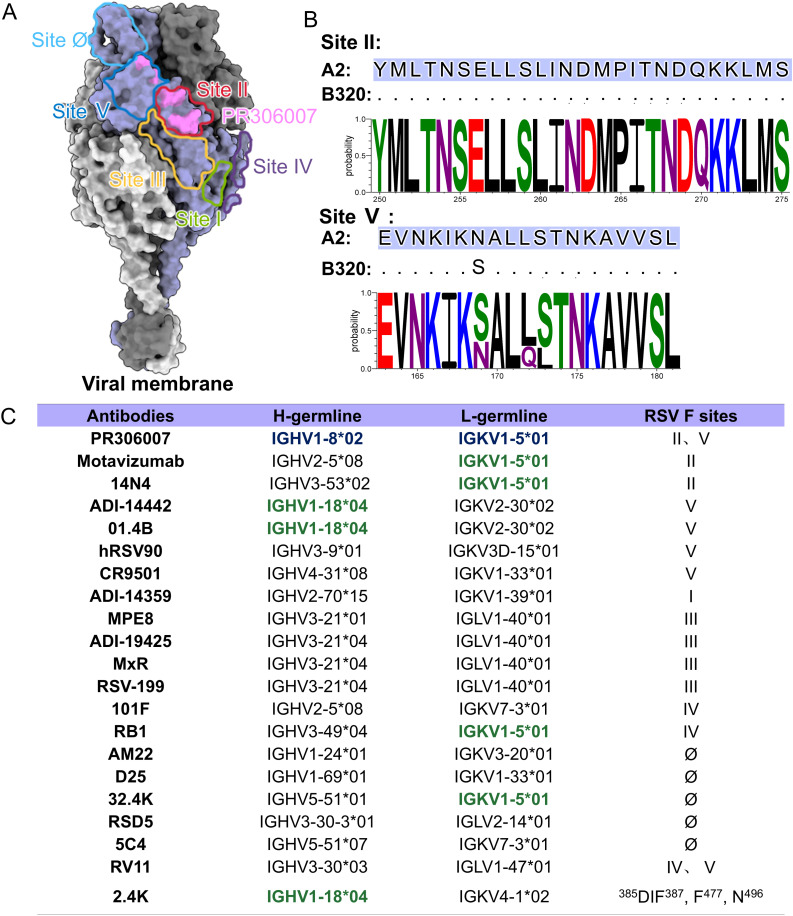
Comparison of PR306007 with previously published antibodies against RSV. (A) The pre-F structure comprises three protomers displayed as surfaces, colored grey, dark grey, and purple, respectively. The epitope comparison of PR306007 with six other previously published antibodies on the A2 pre-F trimer: site Ø is depicted as a light blue line, site II as a red line, site III as an orange line, site IV as a dark purple line, site V as a dark blue line, and site I as a green line. The PR306007 epitope is highlighted in magenta. (B) The sequence conservation of 2383 full-length RSV F genes in the last decade was downloaded from NCBI. The percent conservation of each amino acid within the binding region was calculated and represented as color-coded bars: hydrophilic amino acid in red (acidic amino acid) and blue (basic amino acid), neutral amino acid in green and purple, and hydrophobic amino acid in black. F sequences of RSV A2 with a highlighted footprint of PR306007. (C) Details of PR306007 and previously published human antibodies of RSV, including the germline information for the heavy and light chains, as well as their binding sites on the F protein. Antibodies closely related to PR306007 in terms of germline are highlighted in green.

Based on previous research, six neutralizing antigenic sites have been identified within the two conformations of the F protein: sites Ø, V, III, IV, II, and I (refer to the differently colored regions in Fig 4A). To deeper understand into the neutralizing mechanism of PR306007, we compared the epitopes of PR306007 on DS-Cav1 with that of other previously published monoclonal antibodies (Fig 4A). It is observed that the PR306007’s epitope partially overlaps with that of palivizumab (site II) [26], 14N4 (site II) [27] and hRSV90 (site V) [18], but differs from those recognized by nirsevimab (site Ø) [28], 131-2a (site I) [29], MPE8 (site III) [30] and RB1 (site IV) [31] (Fig 4A). PR306007 interacts with both antigenic site II and site V, which may account for its high potency against RSV. To evaluate the evolutionary conservation of the PR306007 binding site, we performed a sequence alignment of the full-length RSV F protein genes available in NCBI over the past decade. The sequence analysis revealed that the majority of amino acids in the PR306007 epitope are highly conserved across all analyzed RSV A and B subtypes, particularly the residues at site II (Fig 4B). Of note, mutations at residues 172 and 173 are present in currently circulating RSV B variants [32] (Fig 4B). The residue that interacts with S173 in PR306007 is serine, characterized by a hydroxyl group (-OH) that can function as either a hydrogen bond donor or acceptor. Therefore, the variability of residue 173 in the F protein (S173L at site V) does not affect the formation of hydrogen (S12 Fig). Overall, these findings underline the profound conservation of the critical residues involved in PR306007 binding and offer insights into the molecular basis of its potent broad-spectrum neutralizing potential.

To further elucidate the neutralization mechanism of the PR306007, we performed gene sequence analysis of the PR306007 and other human antibodies by using IMGT and IgBLAST (Fig 4C). It is noteworthy that the most frequently used germlines in the reported antibody repertoire against RSV include IGHV1–18, IGHV3–21, IGHV1–69, and IGHV3–30 [23] (Fig 4C). The IGHV1–8*02 and IGKV1–5*01 genes encode PR306007, an antibody derived from immunized humanized mice, which exhibits a germline distinct from other reported antiviral antibodies. PR306007 is more closely related to the germline antibodies 01.4B, 2.4K, and ADI-14442, which are encoded by the IGHV1–18*01 and IGHV1–18*04 genes, respectively. Notably, both 01.4B and ADI-14442 interact with the β3-β4 loop and the base of α4 helix of site V, as well as residues 262–265 associated with antigenic site II [33], sharing a similar binding epitope to that of PR306007 (S13 Fig). This suggests that antibodies derived from closely related germlines may recognize similar epitopes [29], a finding that could facilitate the identification of broader-spectrum neutralizing antibody germlines. Antibodies to site II are currently known to be encoded by several different genes and are not biased towards a particular VH germline gene. However, PR306007 does not fully align with the reported lineages that target site II and demonstrates a binding mode that is distinct from previously described antibodies (Fig 4C).

**Fig 5:**
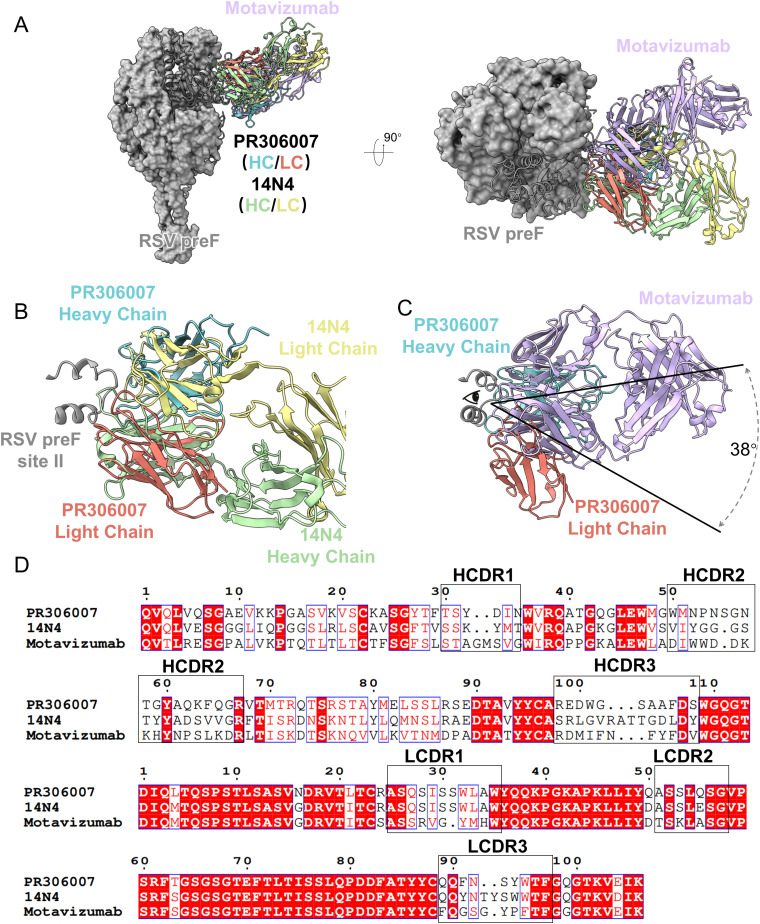
Comparison of known structures of RSV F and the antibodies of site II. (A-C) Structural comparison of the site II antibodies bound to RSV-pre-F: PR306007 (this study), 14N4 (PDB: 5J3D), and motavizumab (PDB: 3IXT). (A) The overall structure is displayed in surface form and rotated 90° in surface form. RSV F is shown as surface presentation colored in grey, PR306007 is shown as cartoon presentation colored in blue (heavy chain, HC) and orange (light chain, LC), and 14N4 in cartoons colored in green (HC) and brick red (LC), and motavizumab in cartoon colored in light purple. (B) Local magnified details of PR306007 and 14N4 binding to the RSV F site II. Despite both having similar regions, these antibodies adopt completely opposite orientations of the HC and LC. PR306007, 14N4, and RSV F site II are shown as cartoons. (C) Local magnified details of PR306007 and motavizumab binding to the RSV F site II, reveal a 38° difference in their binding angles.

The antigenic site II of the RSV F protein is divided into two neutralizing sites: site IIa, which binds to 14N4, and site IIb, which binds to motavizumab. Even though both antibodies target antigenic site II, motavizumab binds RSV F at an angle 42° away from the binding angle of 14N4 [27]. In comparison with the structures of all currently known antibodies with site II, slight differences were observed in the structure of PR306007 (Fig 5A). Regarding binding, PR306007 is more similar to 14N4 and the epitopes partially overlap at the RSV F site II. PR306007 and 14N4 share residues N262, D263, and N268 in terms of binding to antigenic site II, and PR306007 interacts with buried at the base of the helix–loop–helix motif, with the main residues being I266, T267, and D269. Interestingly, PR306007 and 14N4 engage RSV F in similar regions, but adopt absolute opposite HCDR and LCDR orientations (Fig 5B). Compared to motavizumab, PR306007 exhibits a greater difference in binding angle, it is shifted 38° between the two antibodies, but also shares the residues N262 and N268 in the binding epitope (Fig 5C).

### *In vivo* prophylactic efficacy evaluation of PR306007

**Fig 6:**
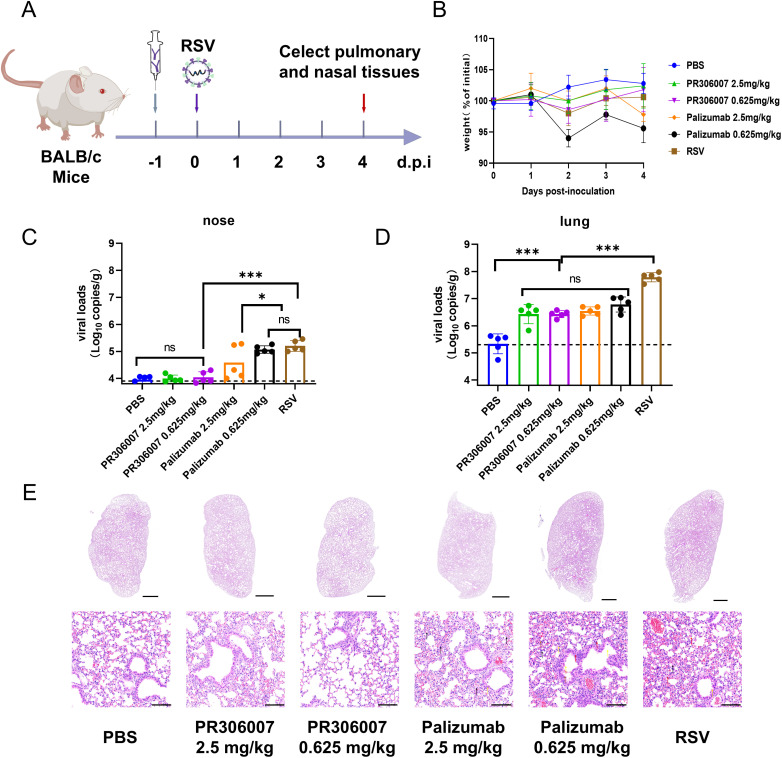
Prophylactic efficacy of PR306007 in RSV–infected mice model. (A) Experimental design for PR306007 neutralization activity testing in Balb/C mice. (B) Body weight changes in mice. For prophylactic efficacy testing, mice were intraperitoneally injected with PBS, 2.5 mg/kg, or 0.625 mg/kg of PR306007 or palivizumab (n = 5 per group) at 24 hours before RSV infection. All groups except the PBS group were infected to RSV A2 stain. Weight changes were monitored daily until 4 days post-infection (d.p.i.). (C, D) Viral load in the respiratory tissues (including nose and lung lobes) collected at necropsy on 4 days post-infection was tested by qRT-PCR. Dashed lines indicate the limit of detection. Data were represented as the mean±S.D. from three biologically independent experiments (n = 3). Significance is determined by one-way ANOVA. *P < 0.05, **P < 0.01, ***P < 0.001; ns, not significant. All the data for this figure can be found in S5 and S6 Tables. (E) Histopathological analyses of mice. Representative images of lung sections stained by Hematoxylin–eosin(HE) infected with RSV at 4 d p.i.. Data are mean±SEM. Black, yellow, and red indicate pathological changes in the alveoli, bronchi/bronchioles, and inflammatory cell infiltration, respectively. The images on the top (bars = 1000 μm) are enlarged regions show a partially enlarged area depicted in the bottom images (bars = 100 μm).

To evaluate the potential clinical application of PR306007, we further assessed its *in vivo* prophylactic efficacy against RSV infection in Balb/C mice model [17,34–36]. Eight-week-old female mice were randomly divided into six groups (n = 5), receiving two doses of PR306007 via intraperitoneal injection: low dose (0.625 mg/kg) and high dose (2.5 mg/kg). Palivizumab was included as a comparative control [17], and the RSV group and blank group received phosphate-buffered saline (PBS). Except for the blank group, mice were intranasally inoculated with RSV A2 virus (1 x 10^5^ PFU per mouse) 24 hours after administration of antibodies or PBS. After four days post-infection, nasal and lung tissues from mice were collected for viral loads, and pulmonary histopathology assessment (Fig 6A).

Viral loads analysis showed that both two doses of PR306007 effectively prevented RSV A2 infection in both the upper (nasal turbinates) and lower (lungs) respiratory tracts of mice by quantitative real-time RCR (qRT-PCR). In the nasal turbinates, the low dose of PR306007 fully inhibited viral replication, comparable to even superior to those of palivizumab (Fig 6C). In the lungs, the low dose of PR306007 also effectively suppressed viral replication, yielding comparable to palivizumab (Fig 6D). These results indicate the preventive potency of PR306007 against RSV A2 infection in the Balb/C mice model.

Additionally, hematoxylin-eosin (HE) staining showed that lung tissues from the RSV group exhibited significant pulmonary inflammation, along with minor lung lesions and hemorrhage (Fig 6E). In contrast, lung tissue from mice pretreated with both two doses of PR306007 showed normal structural integrity, with no significant infiltration of inflammatory cells observed (Fig 6E). Taken together, these results suggest similar prophylactic efficacy of PR306007 to palivizumab against RSV infection, indicating its great clinic potential for RSV prevention.

## Discussion

Passive immunization using RSV F antibodies is a mainstream prevalent strategy for protecting infants and children from RSV infections. To date, various RSV antibodies isolated recovered individuals or mice subsequently humanized have progressed to clinical stages [37]. However, neutralizing antibodies with robust autologous neutralization activity may easily exhibit reduced effectiveness against future variants, and these antibodies also exhibit certain limitations [37,38]. In this study, we immunized Harbour H2L2 transgenic mice with RSV F protein and obtained antibody sequences using the Berkeley Lights Beacon system. We identified a novel and effective antibody, PR306007. PR306007 preferentially binds to the RSV pre-F conformation, with its unique epitope primarily compassing the conserved site II while also interacting with site V. In contrast to other antibodies that target site II, such as motavizumab and 14N4, PR306007 interacts with the F glycoprotein at a distinct angle and with opposite CDR orientations. Previous studies have shown that epitopes Ø and V on the pre-F protein can elicit stronger nAbs responses [17], suggesting that the potent neutralizing activity of PR306007 may be related to its interaction with site V. Moreover, there is a risk of variations at the V site of the pre-F protein. For instance, the substitutions L172Q and S173L in RSV B variants resulted in the ineffectiveness of REGN222 (suptavumab) during phase III trials [32]. Although PR306007 interacts with residues S173 and N175. It is noteworthy that N175 is conserved across various RSV B strains. The residue that interacts with S173 in PR306007 is serine, which possesses a hydroxyl group (-OH) capable of acting as either a hydrogen bond donor or acceptor. This implies that the S173L mutation is unlikely to disrupt hydrogen bond formation, thereby indicating a significant degree of conservation at the binding site. Previous studies have shown that the II site on the F protein is highly conserved across both RSV A and B variants [39]. The remarkable conservation of site II, coupled with the potent neutralizing activity conferred by site V, facilitates PR306007 in achieving both broad-spectrum and high potency, thereby reducing the likelihood of viral escape. The identification of variants resistant to the neutralization efficacy of PR306007 necessitates further investigation into the isolation of its resistant mutations under selective pressure from PR306007. Additionally, it should be noted that PR306007 displays a higher off-rate (Kd), potentially attributed to a smaller interaction area, resulting in lower dissociation energy. Targeted modifications to address this aspect could enhance the antibody’s affinity and protective capability *in vivo*. With the advancement of AI computational methods, the optimization design of neutralizing antibody based on structure represents the rapid and effective strategy for obtaining a promising potent broad-spectrum nAb against RSV.

In comparison to previously reported antibodies, the majority of those screened from humanized mice predominantly belong to the VH1, VH3, and VH4 families. Notably, they also encompass lineages such as IGHV1–8, which are relatively rare within the human antibody repertoire. This highlights the diversity of their genetic lineages. Considering that IGHV1–8 and IGHV1–18 share high sequence similarity, we compared the primary sequence and three-dimensional structure of PR306007 those of 2 other nAbs [33,40] from IGHV1–18 (S13 Fig). Despite the high similarity of their HCDRs, alignment of the three-dimensional complex structures revealed different binding modes with each antibody binding RSV F distinct epitope. Therefore, a cocktail with combination of PR306007 and previous antibody isolated from convalescent individuals may contribute to maintain efficacy against evolving viral strains in the future and to reduce the potential risk of the resistant variants associated with the use of single mAb.

The recognition and interaction between viruses and host cell receptors are crucial for the initial establishment of infection. Several functional receptors of RSV have been identified, and the role of nucleolin (NCL) in mediating endocytosis has been elucidated as a pathway for its infection [41,42]. However, the molecular mechanisms of its binding and membrane fusion remain unclear, which has hindered the development of RSV vaccines and therapeutics. Current research indicates that mAbs that bind to site II do not inhibit viral attachment; rather, they prevent the fusion of the virus with the host cell membrane, although the precise mechanism of action remains unclear. Therefore, investigating the mechanism of action of antibody PR306007 against RSV infection will help elucidate how the virus bind to host cell receptors and membrane fusion, providing vital information for the development of vaccines and therapeutic drugs.

In summary, we utilized H2L2 humanized mice and Beacon Optofluidic system isolated a potent neutralizing antibody PR306007. PR306007 not only demonstrates exceptional neutralizing activity against the RSV A2 strain but also shows efficacy against RSV B. Then, we elucidated the cryo-EM structure of the RSV pre-F and PR306007 Fab complex, revealing a neutralizing molecular mechanism characterized by a unique binding mode. PR306007 effectively targets highly conserved amino acids of site II and site V, highlighting its potential as a broad-spectrum anti-RSV therapeutic. Moreover, PR306007 demonstrated effective prophylactic effect in an animal infection model. These findings provide valuable insights and guidance for the development of therapeutics and vaccines.

## Materials and Methods

### Ethics statement

H2L2 mice immunization experiments were performed according to a protocol approved by the Institutional Animal Care and Use Committee (IACUC) of Harbour Biomed (Ethics Number: WF-20220111100410). The *in vivo* prophylactic efficacy experiments were approved by the IACUC of the Guangzhou National Laboratory (Ethics Number: GZLAB-AUCP-2024-01-A6) and performed in the Animal Biosafety Level 2 (ABSL-2) facility at the Guangzhou National Laboratory.

### Expression and purification of RSV pre-F protein

The sequence of RSV pre-F protein residues 1–513 (DS-Cav1) from the A2 strain (accession no. P03420), with four substitutions S155C/S290C/S190F/V207L) [5], was synthesized and cloned into the mammalian expression vector pCDNA3.1. This construct was fused with the T4 fibritin trimerization domain and a 6 × His tag at the C-terminal. The plasmid was kindly provided by a collaborating laboratory. Plasmid for mammalian codon-optimised RSV F (DS-Cav1) protein was transfected using Expi 293 F cells and proteins were purified from culture supernatants. Specifically, cell culture supernatants were harvested on day 5 after plasmid transfection and RSV F protein was purified using Ni-Sepharose chromatography (GE Healthcare). The protein was further purified on a Superose 6 Increase 10/300 size-exclusion column (GE Healthcare) equilibrated with PBS.

### Harbour H2L2 transgenic mice immunization

H2L2 humanized mice were immunized with the RSV pre-F protein as the immunogen. H2L2 mice were divided into two groups (n = 10) and immunized using different injection methods and adjuvants. One group was passively immunized intraperitoneally, with the primary immunization utilizing Complete Freund’s Adjuvant (CFA) at a dose of 10 μg per mouse. Next, the booster immunizations by injecting 5 µg of antigen with Ribi adjuvant. The other group consisted of 10 µg of antigen emulsified in manganese adjuvant gamma3 delivered to four spots subcutaneously. The interval between each immunization is 2 weeks. Serum was collected one week after each booster immunization, and antibody levels were monitored using ELISA, with a total of 3–4 booster immunizations conducted. Finally, the mice were euthanized, and bone marrow, spleen, and lymph nodes were harvested for B cell processing.

### ELISA serum characterization

A 96-well plate was coated with 100 ng of RSV F protein per well in PBS overnight at 4 °C. Following this, the wells were filled with 200 μL of blocking buffer (PBS pH 7.3, 0.05% v/v Tween-20) and incubated at 37 °C for 1 hour. Mouse sera were serially diluted in the blocking buffer, and the diluted antibodies were added to the wells, followed by incubation at 37 °C for an additional hour. Peroxidase AffiniPure Goat Anti-Rat IgG (H + L) antibody (Jackson, 112-035-062) was diluted 1:5000 in blocking buffer, and 100 μL of this solution was added to each well, followed by incubation at 37 °C for 1 hour. The substrate tetramethylbenzidine (TMB) (BioLegend, 421101) was added at a volume of 50 μL per well and incubated at room temperature in the dark for approximately 15 minutes. The reaction was terminated by adding an equal volume of ELISA stop solution (Solarbio, C1058), and the absorbance was immediately measured using a multimode plate reader.

### Single B cell screening on Beacon Optofluidic system

Plasma B cells were enriched using the CD138^+^ Plasma Cell Isolation Kit (mouse, Miltenyi Biotec) and the EasySep Mouse CD138 Positive Selection Kit (STEMCELL Technologies). The isolated B cells were then counted and stained for sorting. Subsequently, the cells were incubated with the following antibodies for 30 minutes at 4 °C: PE/Cy7 anti-mouse CD3 (BioLegend, 100220), FITC rat anti-Mouse CD19 clone 1D3 (RUO) (BD, 553785), PE/Cyanine7 anti-mouse CD45R/B220 antibody (BioLegend, 103222), and anti-mouse CD138-APC (BD). The CD3^-^CD19^-^B220_-_CD138^+^ population was sorted using a BD FACSAria Fusion (BD, 558626) according to the manufacturer’s recommended settings. The sorted cells were maintained in a 37 °C incubator with 5% CO2 until further use. As previously described, RSV pre-F specific plasma B cells were identified using the Beacon device (Optofluidic System) [20,35,36]. Briefly, sorted plasma B cells were loaded into the nanopen on an OptoSelect 14K chip. The cells were screened in a 50-minute time-course assay for the secretion of antibodies binding to RSV pre-F-conjugated beads mixed with Alexa Fluor 647 AffiniPure Goat Anti-rat IgG (Fc) (Jackson, 112-605-003). These antigen-specific single plasma B cells were then transferred to an Eppendorf twin. tec PCR Plate 96 RNA purification and reverse transcription RT-PCR of the cell lysates from single B cells was performed using a modified protocol provided by Berkeley Lights.

### Sequence analysis and production of mAb

The heavy and light chains of the antibodies were amplified and sequenced by PCR as previously described [21]. Sequence analysis, including the identification of VH and VL regions, was conducted using IMGT database. The VH and VL sequences were cloned into plasmids containing an IgG1 or relevant light-chain backbone (GenScript) utilizing an internal expression vector. Plasmids were transfected into human embryonic kidney 293T cells (ATCC) in 24-well plates using PEI (Polysciences, 23966) and incubated at 37 °C with 5% CO_2_ for 3 days. Supernatants were then collected for subsequent testing. Positive clones identified in the supernatant assays were subsequently transfected into HEK 293F cells (Gibco, R79007) or ExpiCHO cells (Thermo Fisher Scientific, A29127) for small-scale recombinant IgG production. Recombinant IgG was purified using a Protein A Resin (GenScript). For Fab production, heavy-chain plasmids encoding only the VH and CH1 (domain 1 of the heavy-chain constant region) were synthesized and co-transfected with light-chain plasmids into Expi293 cells. A 6-histidine tag was added to the C-terminus of the VH for purification, and the Fabs were purified using a His-tag affinity column.

### Neutralization test of Cell Supernatants

The cell supernatant containing neutralizing antibodies was serially diluted in three-fold dilutions across six gradients and added to a 96-well plate. Then, 100 TCID_50_ of recombinant RSV A Long strain expressing luciferase was added to each well. The plate was incubated at 37 °C in a 5% CO₂ incubator for 1 hour. Meanwhile, 293T cells were digested with trypsin, counted, and resuspended to a concentration of 5 × 10^5^ cells/mL. The diluted cell suspension (100 µL per well) was then added to the 96-well plate, which was subsequently incubated at 37 °C in a 5% CO₂ incubator for 48 hours. After 48 hours, 100 µL of supernatant was carefully removed from each well and replaced with 100 µL of PE luminescent substrate. The plate was left undisturbed for 2 minutes before luminescence was measured using a chemiluminescence reader.

### Neutralization test

In brief, HEp-2 cells were seeded in 24-well plates (2 × 10^5^ cells/well) 16 h before the infection experiment. Antibodies were diluted in DMEM containing 2% FBS (heat inactivated) starting at indicated concentration followed by 4-fold or 3-fold serial dilutions. Antibody dilutions were mixed with 100 pfu of RSV A2 strain or RSV B9320 strain and incubated for 2 h at 33 °C. Following the 2 h incubation, the mixture was added to the plate and incubated at 37 °C for 3–4 days. Cells were observed for plaque formation after 3–4 days of incubation, and plaque counts were compared to virus controls. Alternatively, the cells were harvested, fixed, and permeabilized after incubation. The cells were then incubated overnight at 4 °C with a 1:100 dilution of Respiratory Virus Antibody-FITC (Abcam, ab20391). Following two washes, fluorescent images were acquired using an Evos M5000 Cell Imaging System (Thermo Fisher Scientific) at a magnification of 4X. The fluorescence dots were quantified and normalized based on the total number using ImageJ. The amount of fluorescence is used to determine RSV infection. The inhibition of antibodies and the value of half-maximal neutralizing titers (NT_50_) were calculated as the mean of three independent experiments. Finally, neutralizing titers were analyzed by non-linear regression curve fit in GraphPad Prism.

### ELISA binding assay

96-well plates were coated with 1 μg/mL of either the RSV pre-F or post-F protein in PBS per well and incubated overnight at 4 °C. After following a standard washing and blocking protocol, 100 μL of a 1 μg/mL mAb was added to each well. Then, 100 μL of a 10-fold serially diluted antibody was introduced to each well, and the plates were incubated for 1 hour at 37 °C. The wells were washed five times afterward. Following this, 100 μL of horseradish peroxidase (HRP)-conjugated anti-human IgG Fc antibody (Abcam, ab99759) diluted to 1:2000 was added to each well and incubated for 1 hour at 37 °C. After washing again, 100 μL of TMB substrate (Solarbio, PR1200) was added and incubated for 15 minutes. The absorbance at 450 nm was measured immediately after the stop solution (Solarbio, C1058) was added. The half-effective concentration (EC_50_) was determined using sigmoidal curve fitting in GraphPad Prism software (GraphPad Software, CA).

### Antibodies binding kinetics measured by SPR

Surface plasmon resonance. All surface plasmon resonance (SPR) experiments were performed on a Biacore 200 instrument (GE Healthcare). To study antibody binding to RSV F, a trimeric RSV F glycoprotein stabilized with a fibritin trimerization moiety was covalently coupled to 135 response units (RUs) CM5 chip, and a blank surface without antigen was generated under the same coupling conditions as a reference. PR306007 Fab was diluted 2-fold to a starting concentration of 1.5625 nM in 10 mM HEPES (pH 7.5), 150 mM NaCl, and 0.05% polysorbate 20 (HBS-EP) and then injected into the immobilized F-glycoproteins and reference cells at 50 μL/min. At a flow rate of 100 μL/min, the surface was regenerated with 100 μL of 10 mM glycine (pH 1.5). Data were processed with SCRUBBER-2 and double referenced by subtraction of the blank surface and blank supply (no analyte). Binding curves were fitted globally to a 1:1 binding model, and the equilibrium dissociation constant K_D_ was calculated by dividing the dissociation rate constant by the binding rate constant.

### Negative-stain electron microscopy

The purified RSV pre-F protein or RSV pre-F-PR306007 complex of 5 μL was applied to the grid and allowed to incubate for 30 seconds. Following this, the grid was stained with 100 μL of a 3% (w/v) uranyl acetate solution for 40 seconds. Images were captured using a Thermo Scientific Tecnai Spirit T20 (FEI) microscope, which was operated at 120 kV and equipped with an FEI Ceta 16M camera (4 k × 4 k) at 92,000 magnification at Guangzhou National Laboratory.

### Preparation of cryo-EM specimen

C-flat 1.2/1.3 on 200 gold mesh were glow discharged (Pelco easiGlow) for 23 s at 25 mA. For the RSV F: PR306007 Fab complex, RSV F at 0.8 mg mL^−1^ was mixed with the PR306007 Fab at a 1: 1.2 molar ratio. The mixture (3 µL) was applied to the grid and immediately plunged into liquid ethane using an FEI Vitrobot Mark IV (FEI ThermoFisher) after blotting for 5 s, blot force at 0, at 22 °C and ~100% relative humidity.

### Cryo-EM data collection

The samples were collected using a 300 kV with a FEI Titan microscope (Thermo Fisher) equipped with a direct electron detector (Falcon III, FEI). Micrograph images were collected using the parameters including movies (32 frames, 0.2 s), total dose of 60 e − Å^−2^, a defocus range between -1.8 to -1.2 μm and recorded at a calibrated magnification of ×22,500X, thus resulting in a pixel size of 1.07 Å per pixel. Data collection and refinement statistics are summarized in S4 Table.

### Image processing and three-dimensional reconstruction

The cryo-EM data set was processed primarily in cryoSPARC [43]. Individual frames from each micrograph movie were aligned and averaged using MotionCorr2 [44] to produce drift-corrected images. Particles were picked and selected to allow template picking and contrast transfer function (CTF) was estimated using parch CTF estimation integrated in cryoSPARC. A few rounds of Ab initio and heterogenous were used to classify the particles and test symmetry further, then run Local Refinement. Until the map quality and resolution could no longer be improved by further reducing the particle numbers, a final nonuniform refinement step was used. For all reconstructions, the final resolution was assessed using the gold standard FSC (FSC = 0.143) criterion [43].

### Model building and refinement

The X-ray crystal structures of the Fab (PDB code: 8F9U) and RSV pre-fusion protein (PDB code: 4MMV) were manually placed into the cryo-EM density map for the complex of pre-F and Fab mature particles data set and rigid-body fitted with UCSF Chimera [45]. The fitting was further improved with real-space refinement using Phenix [46]. Manual model building was performed using Coot [47] and in combination with real space refinement with Phenix [46]. Only atomic coordinates were refined and the maps were kept constant. The validation was reported by the MolProbity [48] function integrated within Phenix. Refinement statistics are shown in S4 Table.

### Prophylactic *in vivo* BALB/c challenge study

Groups of five 8-week-old female BALB/c mice were passively immunized intraperitoneally with PR306007, palivizumab, or PBS. Antibodies were administered at two doses: a low dose (0.625 mg/kg) and a high dose (2.5 mg/kg). Under isoflurane anesthesia, mice were administered 100 µL of antibody or PBS on Day 1. The PBS group served as the negative control, while the RSV-inoculated group acted as the positive control. On Day 2, approximately 24 ± 2 hours after antibody injection, mice were intranasally inoculated with 50 µL of 10^5^ pfu RSV A2 virus under anesthesia, except for the PBS group, which did not receive viral inoculation. Body weights were monitored daily, and the animals were humanely euthanized on Day 4 post-inoculation for tissue collection.

### qRT-PCR

Lung tissues were weighed and homogenized in 1 mL of TRIzol reagent (Thermo Fisher, 15596018CN), and RNA was extracted according to the manufacturer’s protocol. Briefly, qRT- PCR was performed using the HiScript II One Step qRT-PCR SYBR Green Kit (Vazyme, Q221), following the instructions provided. The thermal cycling conditions were as follows: 50 °C for 15 minutes, 95 °C for 30 seconds, followed by 40 cycles of 95 °C for 10 seconds and 60 °C for 30 seconds. Primers were designed to target the F gene of RSV A2: RSV F-F: 5′-CGAGCCAGAAGAGAACTACCA-3′ and RSV F-R: 5′-CCTTCTAGGTGCAGGACCTTA-3′.

## Supporting information

S1 Fig Detection of antibody titers in serum of H2L2 mice after immunization. (A-D) This corresponds to the 1^st^ to 4^th^ blood draws, respectively. The left group was immunized using Freund’s adjuvant and intraperitoneal injection, while the right group was immunized with manganese adjuvant and subcutaneous multi-site injections. Sera were initially diluted at 1:100 and 1:1000, followed by threefold dilutions up to 1:181,000. Based on the binding affinity of serum antibodies to RSV pre-F, six mice (designated as 606, 607, 608, 610, 617, and 619) were selected for further experiments.
(TIF)

S2 Fig Flow cytometry results of plasma B cell isolation from bone marrow (A) and spleen and lymph node (B). CD3 is a specific marker for T cells; CD19 is a pan-B cell marker, expressed at low levels or not at all on plasma cells; CD45R is expressed on mouse B lineage cells, except for plasma cells; CD138 is highly expressed on plasma cells. The cells of CD3^-^CD19^-^CD45R^-^CD138^+^ are plasma B cells.
(TIF)

S3 Fig Phylogenetic tree of the 78 selected antibody sequences. The red dots highlight the 12 antibodies with superior neutralizing activity. The bar representing 0.05 indicates the degree of genetic variation among each antibody.
(TIF)

S4 Fig Neutralization assay results of cell supernatants containing antibodies. The neutralization assay results for the cell supernatants of 78 antibodies were obtained as follows: The cell supernatants containing antibodies were diluted threefold and mixed with a recombinant RSV A Long strain expressing luciferase. The mixture was then added to 293T cells and incubated for 48 hours. The ID_50_ values were calculated based on the dilution factors. The blue dashed line represents the log value of 1.6.
(TIF)

S5 Fig Expression and purification result of the selected 12 antibodies. The two target bands on the gel correspond to the HC and LC of the antibody on the SDS-PAGE profile.
(TIF)

S6 Fig Binding assays of three antibodies (PR306007, MEDI-8897, and palivizumab) for RSV pre-F. (A-C) These antibodies (PR306007, MEDI-8897, and palivizumab) were tested for their capability to bind the RSV pre-F by Biacore sensorgrams in triplicate experiments, along with their respective KD, Kon, and Koff values. Palivizumab and MEDI-8897 served as controls.
(TIF)

S7 Fig Characterization of RSV pre-F and PR306007 Fab: pre-F complex (A and B). Left: Purification of RSV pre-F (A) and Fab PR306007: pre-F complex (B) by size exclusion chromatography. Right: SDS-PAGE for the complex of RSV pre-F (A) and PR306007 Fab: pre-F complex (B). Purified RSV F protein was analyzed for purity by SDS-PAGE under reducing conditions to disrupt intra-protein disulfide bond between F1 and F2 subunit.
(TIF)

S8 Fig Negative stain-electron microscopy of RSV pre-F(A) and RSV pre-F in complex with PR306007 Fab(B). All scale bars are 100 nm.
(TIF)

S9 Fig Structure refinement workflow of RSV pre-F in complex with PR306007 Fab. (TIF)

S10 Fig The resolution determination of cryo-EM reconstruction. (A) A local-resolution presentation. (B) The gold-standard FSC. (C) Histogram and Directional FSC Plot.
(TIF)

S11 Fig Representative density maps. The cartoon model of PR306007: pre-F complex is superimposed with a semitransparent surface. In the surrounding boxes, atomic models shown as either stick are superimposed to indicate the representative regions in wireframes. PR306007 heavy chains colored sky blue. PR306007 light chains colored orange. In the stick models, amino acid residue numbers are indicated.
(TIF)

S12 Fig Analysis of the impact of the non-conservative characteristics of amino acid at RSV F 173 on PR306007 binding. (A) Conservative analysis of site V. The sequence conservation of 2383 full-length RSV F genes in the last decade was downloaded from NCBI. Related to Fig 4B. (B) Close-up views of the interaction between S173 of RSV pre-F and S67 of the PR306007 VL domain. (C) Close-up views of the interaction between L173 mutation of RSV pre-F and S67 of the PR306007 VL domain. The VL of the PR306007 and a pre-F protomer are depicted as cartoons, in orange (light chain) and purple (pre-F), respectively. For clarity, key contact residues are labeled and displayed as sticks, with oxygen atoms in red and nitrogen atoms in blue. Hydrogen bonds are represented by black dashed lines.
(TIF)

S13 Fig Sequence alignment of selected nAbs derived from VH1–18 germline. (A) Alignment of selected NAbs derived from VH1–18 germline. PR306007 (HC: peacock blue, LC: Dark), ADI14442 (HC: teal, LC: Brick red), and 2.4K (pink) are superimposed. RSV F is shown in the surface, a protomer colored purple and other two protomers colored grey. (B) Sequence alignment of the HC variable domain sequence of PR306007 to other reported antibodies from the VH1–18 germline.
(TIF)

S1 Table The germline information of heavy- and light-chain germline gene pairing of 331 antibodies. (XLSX)

S2 Table The individual numerical values for the following figure panel: related to Fig 2B. (XLSX)

S3 Table The individual numerical values for the following figure panel: related to Fig 2D. (XLSX)

S4 Table Cryo-EM data collection, and refinement. (DOCX)

S5 Table The individual numerical values for the following figure panel: related to Fig 6C. (XLSX)

S6 Table The individual numerical values for the following figure panel: related to Fig 6D. (XLSX)
