# Supplementary figures and images for: Discovery and molecular mechanism of potent neutralizing antibody from humanized mice with respiratory syncytial virus
Source: PLoS Pathog. 2025 Nov 17;21(11):e1013674. doi: 10.1371/journal.ppat.1013674 (PMC12637950; doi:10.1371/journal.ppat.1013674)

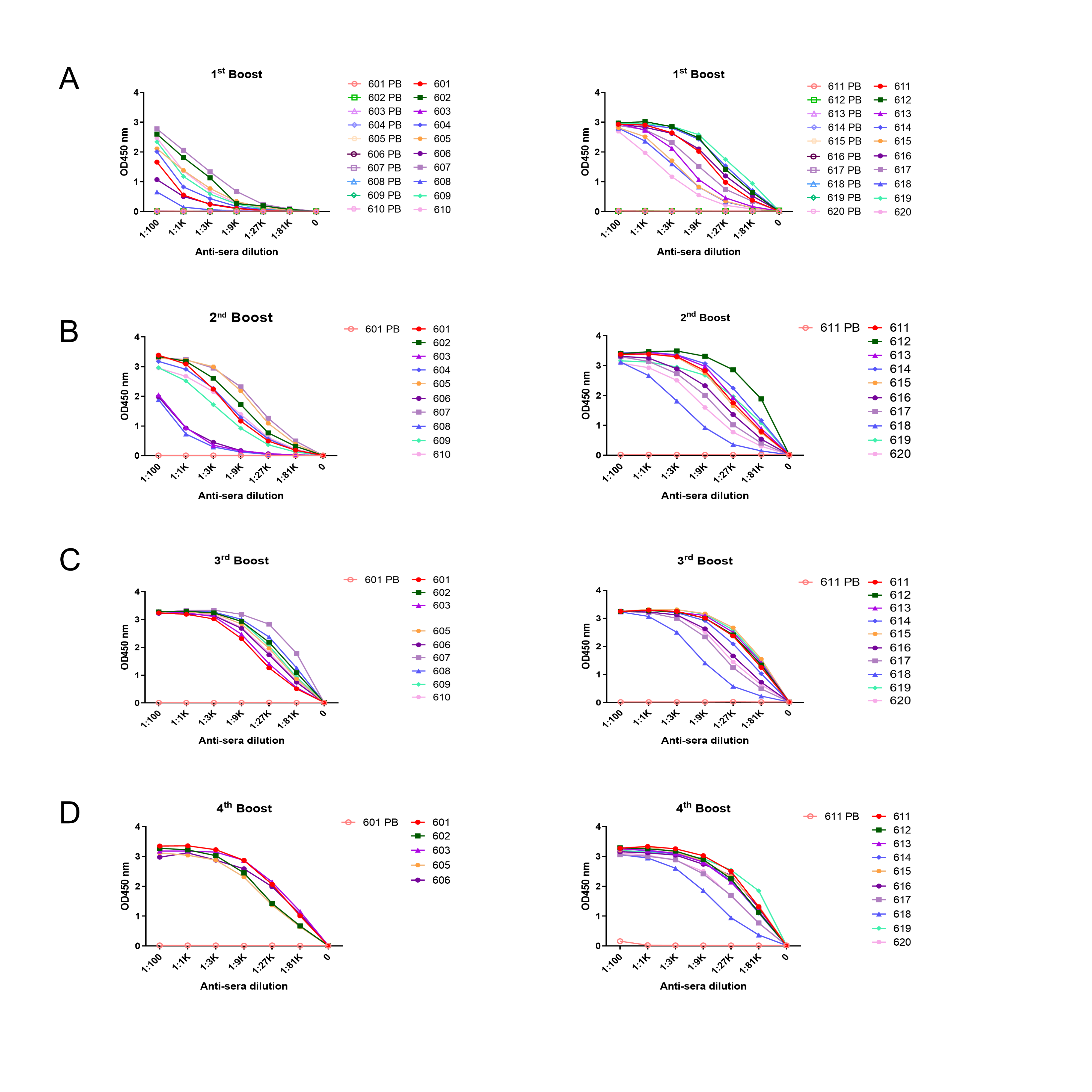

Supplement: S1 Fig — (A-D) This corresponds to the 1st to 4th blood draws, respectively. The left group was immunized using Freund’s adjuvant and intraperitoneal injection, while the right group was immunized with manganese adjuvant and subcutaneous multi-site injections. Sera were initially diluted at 1:100 and 1:1000, followed by threefold dilutions up to 1:181,000. Based on the binding affinity of serum antibodies to RSV pre-F, six mice (designated as 606, 607, 608, 610, 617, and 619) were selected for further experiments. (TIF) [file ppat.1013674.s001.tif]

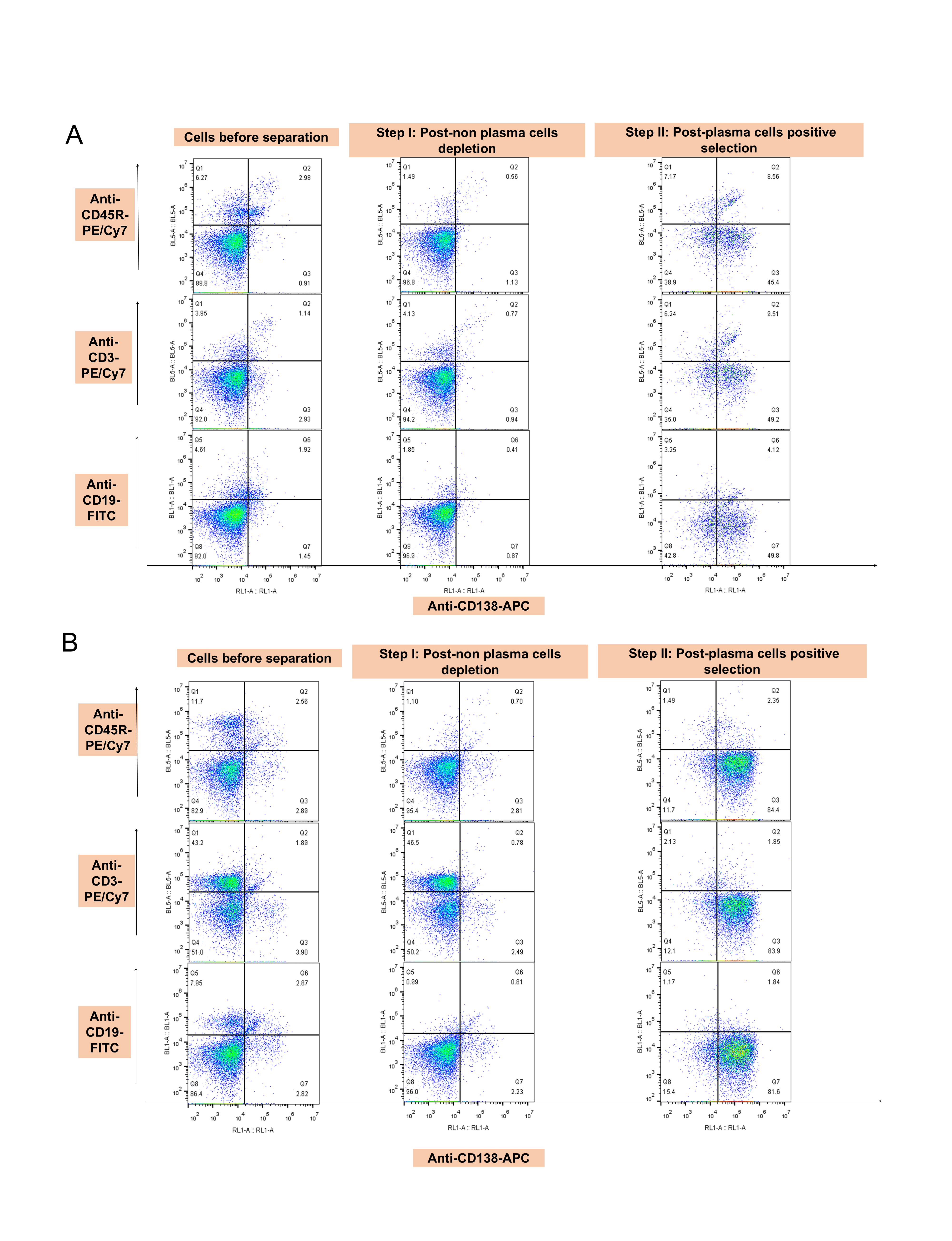

Supplement: S2 Fig — CD3 is a specific marker for T cells; CD19 is a pan-B cell marker, expressed at low levels or not at all on plasma cells; CD45R is expressed on mouse B lineage cells, except for plasma cells; CD138 is highly expressed on plasma cells. The cells of CD3-CD19-CD45R-CD138+ are plasma B cells. (TIF) [file ppat.1013674.s002.tif]

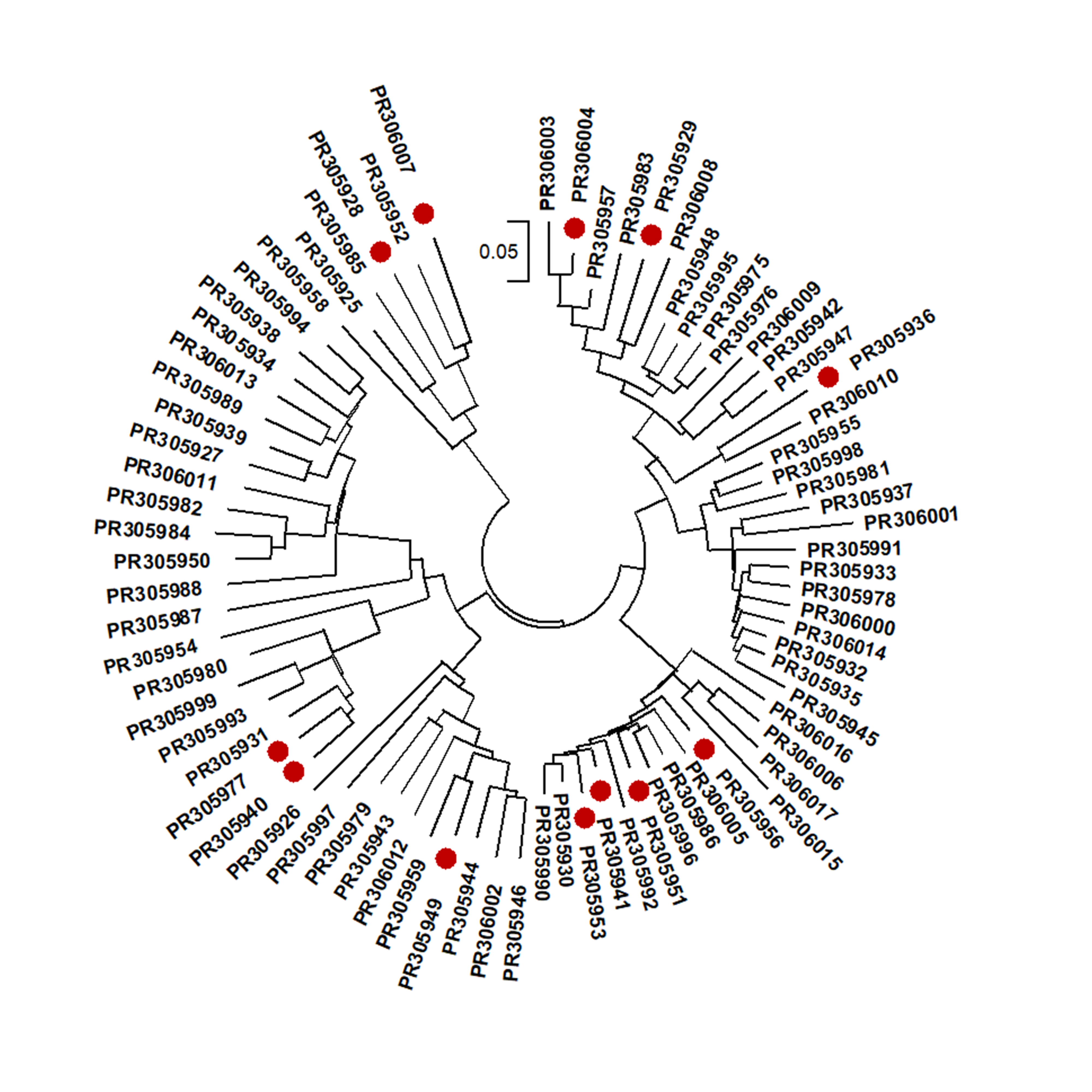

Supplement: S3 Fig — The red dots highlight the 12 antibodies with superior neutralizing activity. The bar representing 0.05 indicates the degree of genetic variation among each antibody. (TIF) [file ppat.1013674.s003.tif]

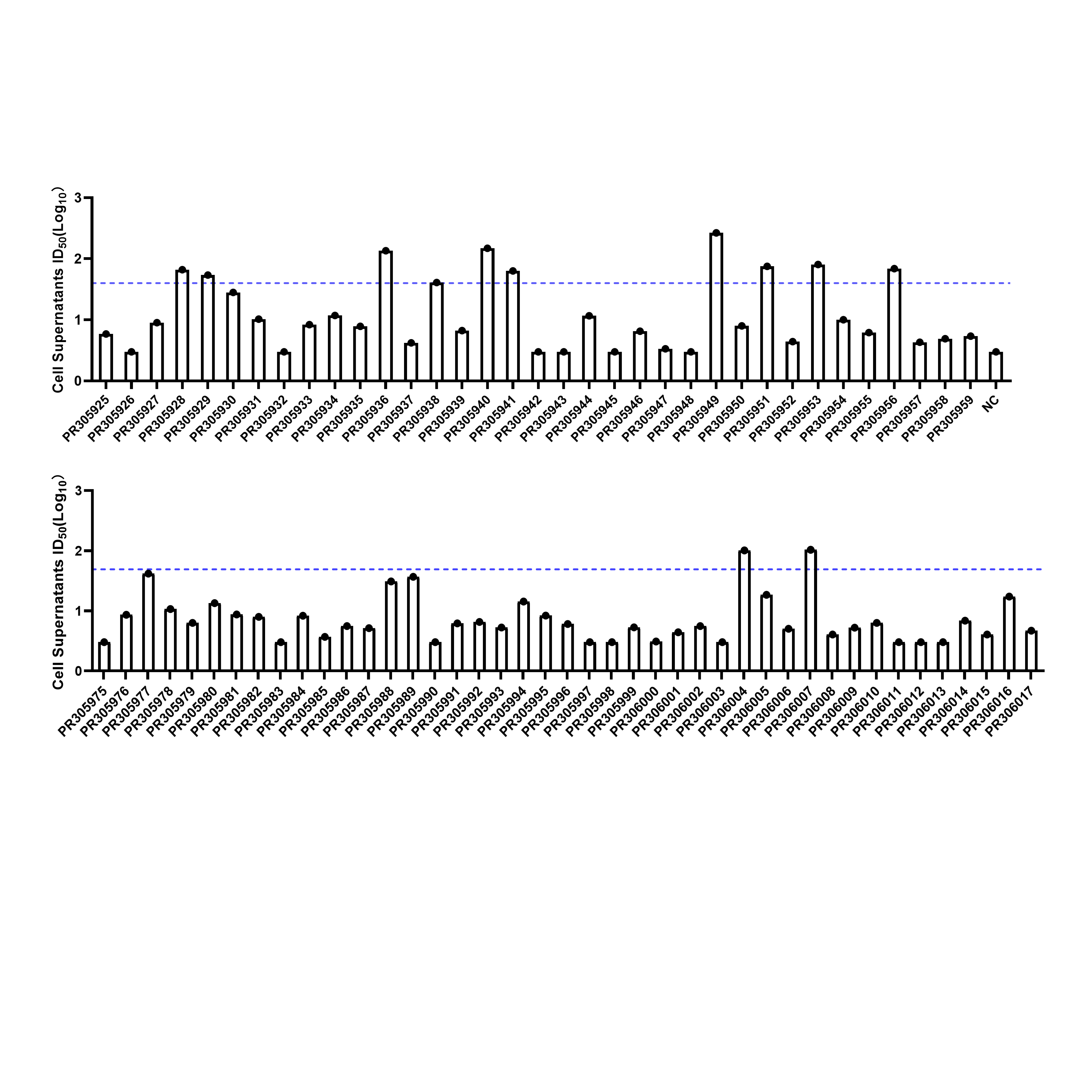

Supplement: S4 Fig — The neutralization assay results for the cell supernatants of 78 antibodies were obtained as follows: The cell supernatants containing antibodies were diluted threefold and mixed with a recombinant RSV A Long strain expressing luciferase. The mixture was then added to 293T cells and incubated for 48 hours. The ID50 values were calculated based on the dilution factors. The blue dashed line represents the log value of 1.6. (TIF) [file ppat.1013674.s004.tif]

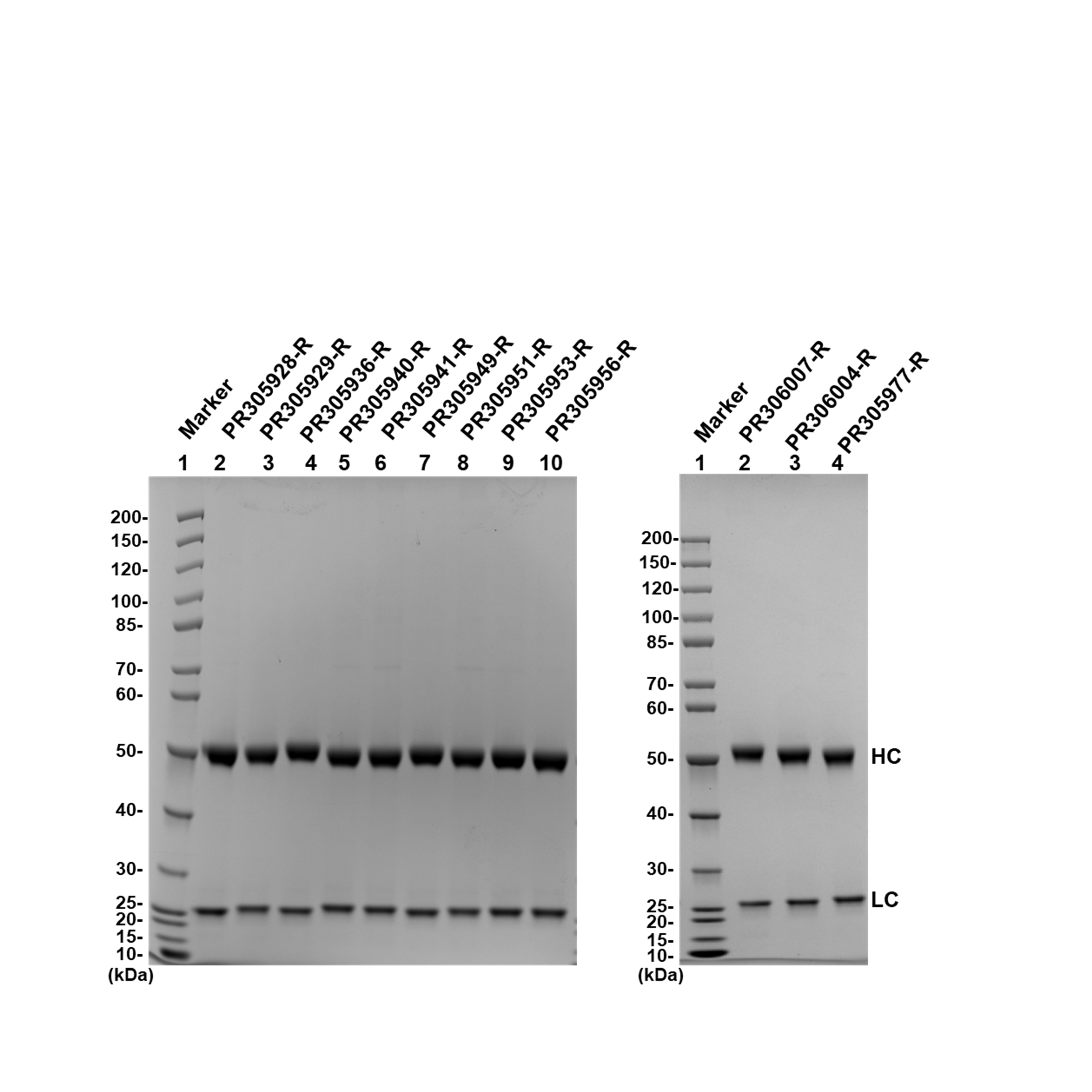

Supplement: S5 Fig — The two target bands on the gel correspond to the HC and LC of the antibody on the SDS-PAGE profile. (TIF) [file ppat.1013674.s005.tif]

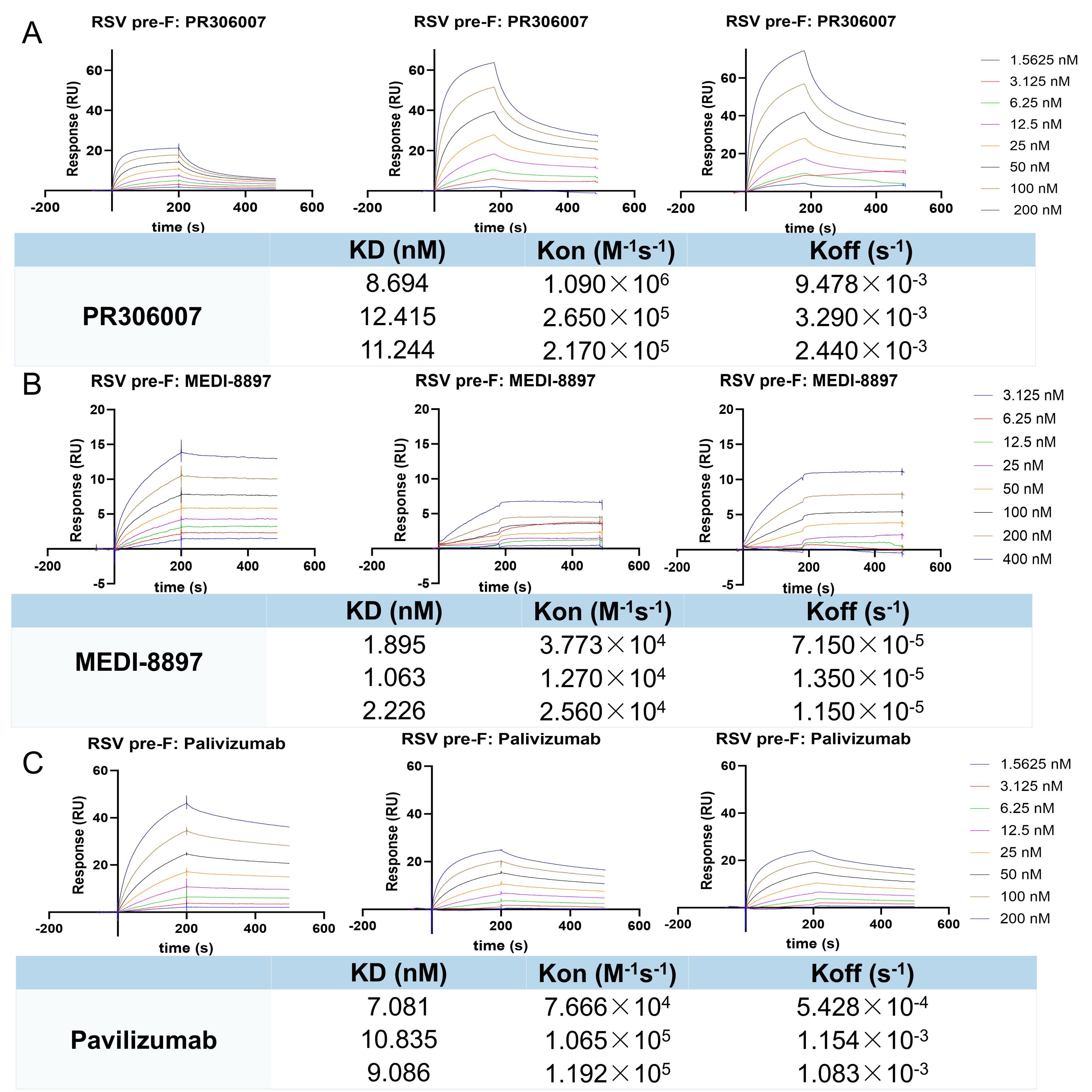

Supplement: S6 Fig — (A-C) These antibodies (PR306007, MEDI-8897, and palivizumab) were tested for their capability to bind the RSV pre-F by Biacore sensorgrams in triplicate experiments, along with their respective KD, Kon, and Koff values. Palivizumab and MEDI-8897 served as controls. (TIF) [file ppat.1013674.s006.tif]

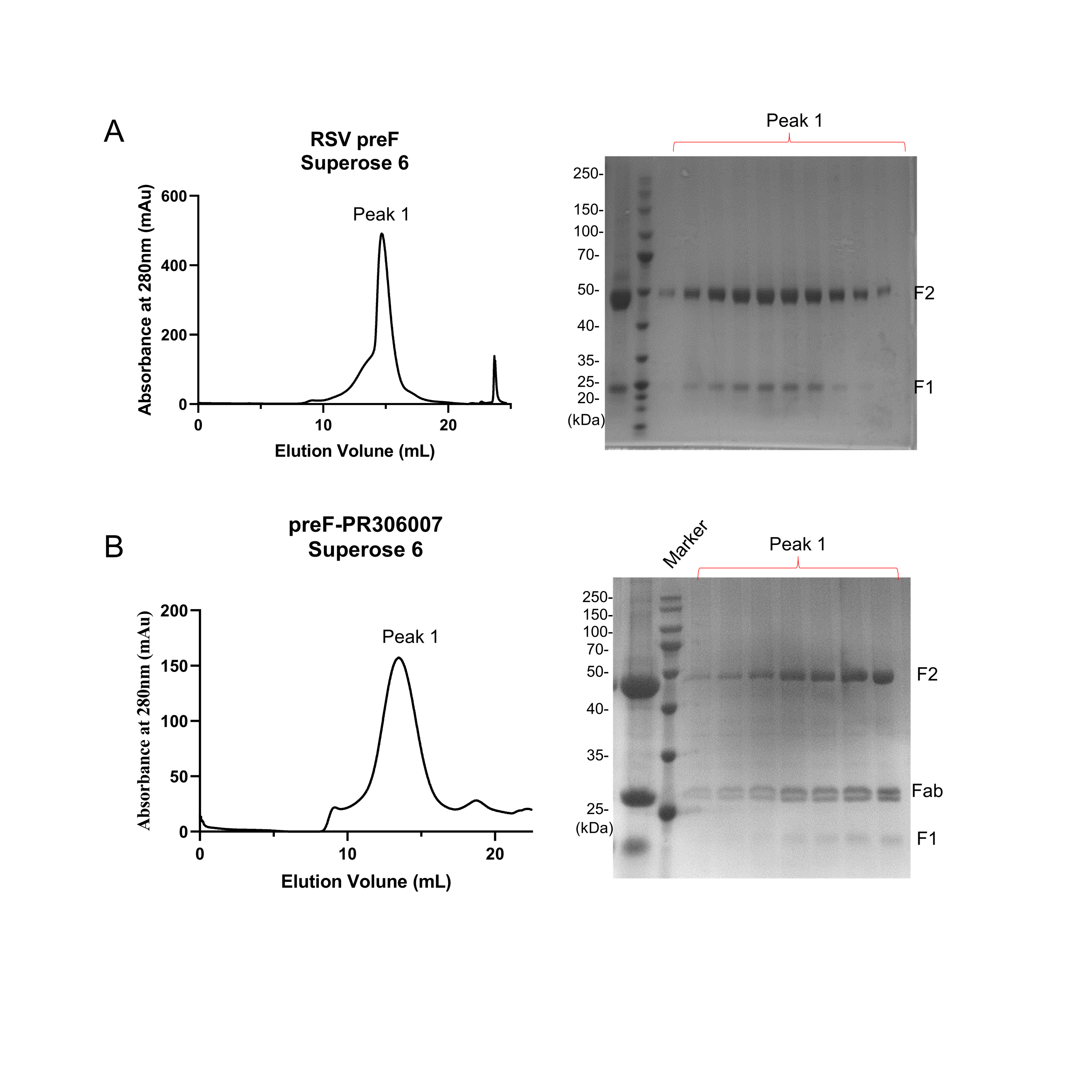

Supplement: S7 Fig — Left: Purification of RSV pre-F (A) and Fab PR306007: pre-F complex (B) by size exclusion chromatography. Right: SDS-PAGE for the complex of RSV pre-F (A) and PR306007 Fab: pre-F complex (B). Purified RSV F protein was analyzed for purity by SDS-PAGE under reducing conditions to disrupt intra-protein disulfide bond between F1 and F2 subunit. (TIF) [file ppat.1013674.s007.tif]

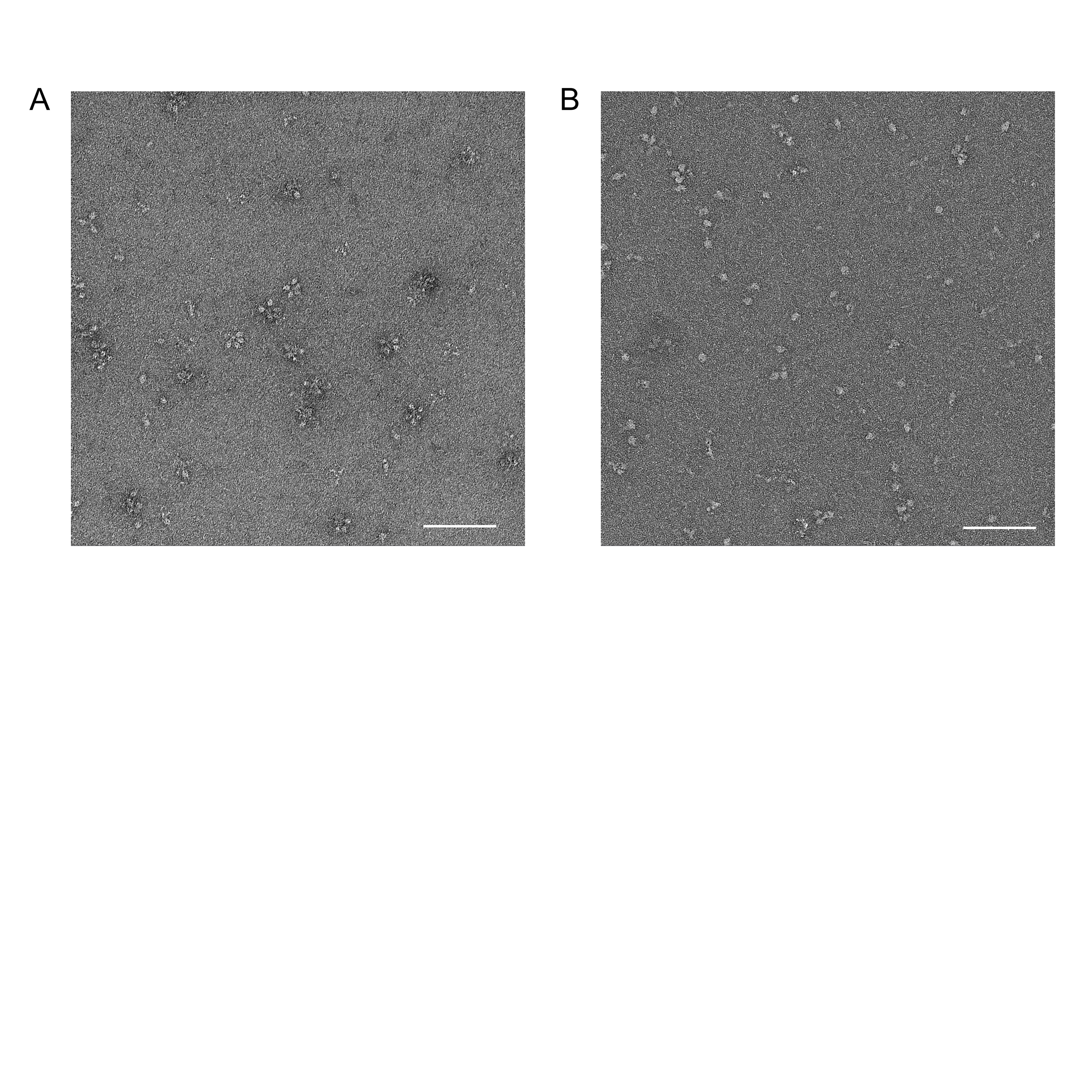

Supplement: S8 Fig — All scale bars are 100 nm. (TIF) [file ppat.1013674.s008.tif]

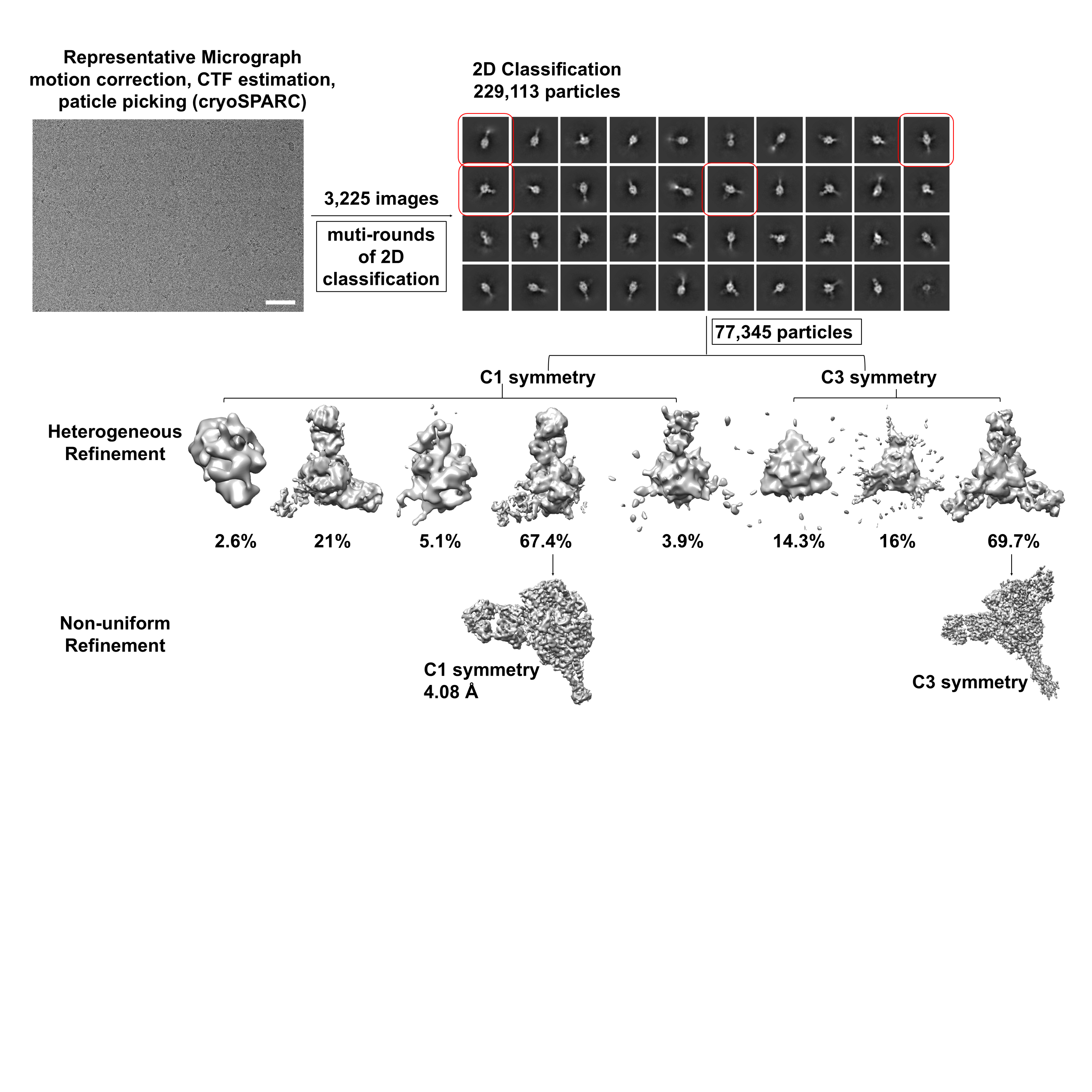

Supplement: S9 Fig — (TIF) [file ppat.1013674.s009.tif]

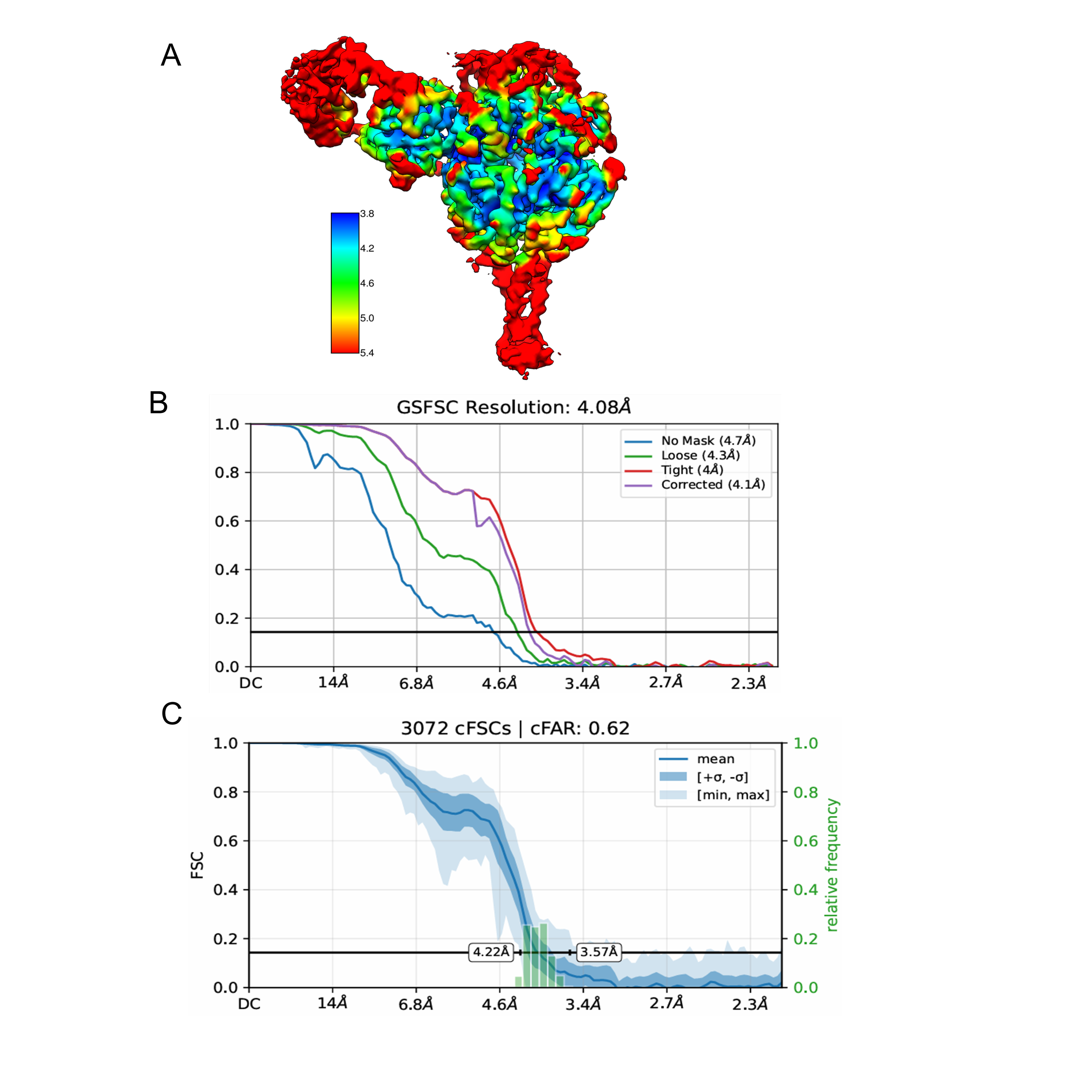

Supplement: S10 Fig — (A) A local-resolution presentation. (B) The gold-standard FSC. (C) Histogram and Directional FSC Plot. (TIF) [file ppat.1013674.s010.tif]

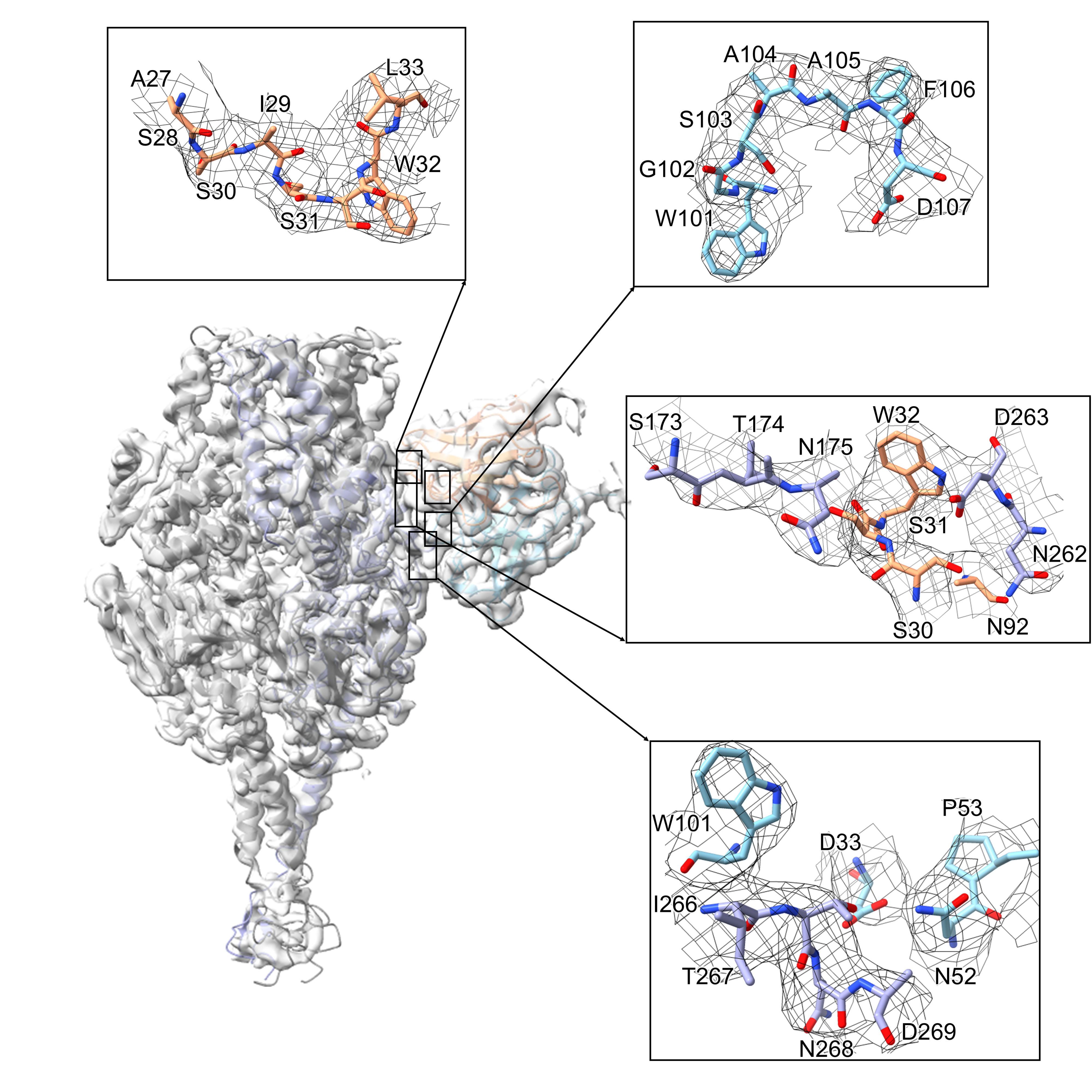

Supplement: S11 Fig — The cartoon model of PR306007: pre-F complex is superimposed with a semitransparent surface. In the surrounding boxes, atomic models shown as either stick are superimposed to indicate the representative regions in wireframes. PR306007 heavy chains colored sky blue. PR306007 light chains colored orange. In the stick models, amino acid residue numbers are indicated. (TIF) [file ppat.1013674.s011.tif]

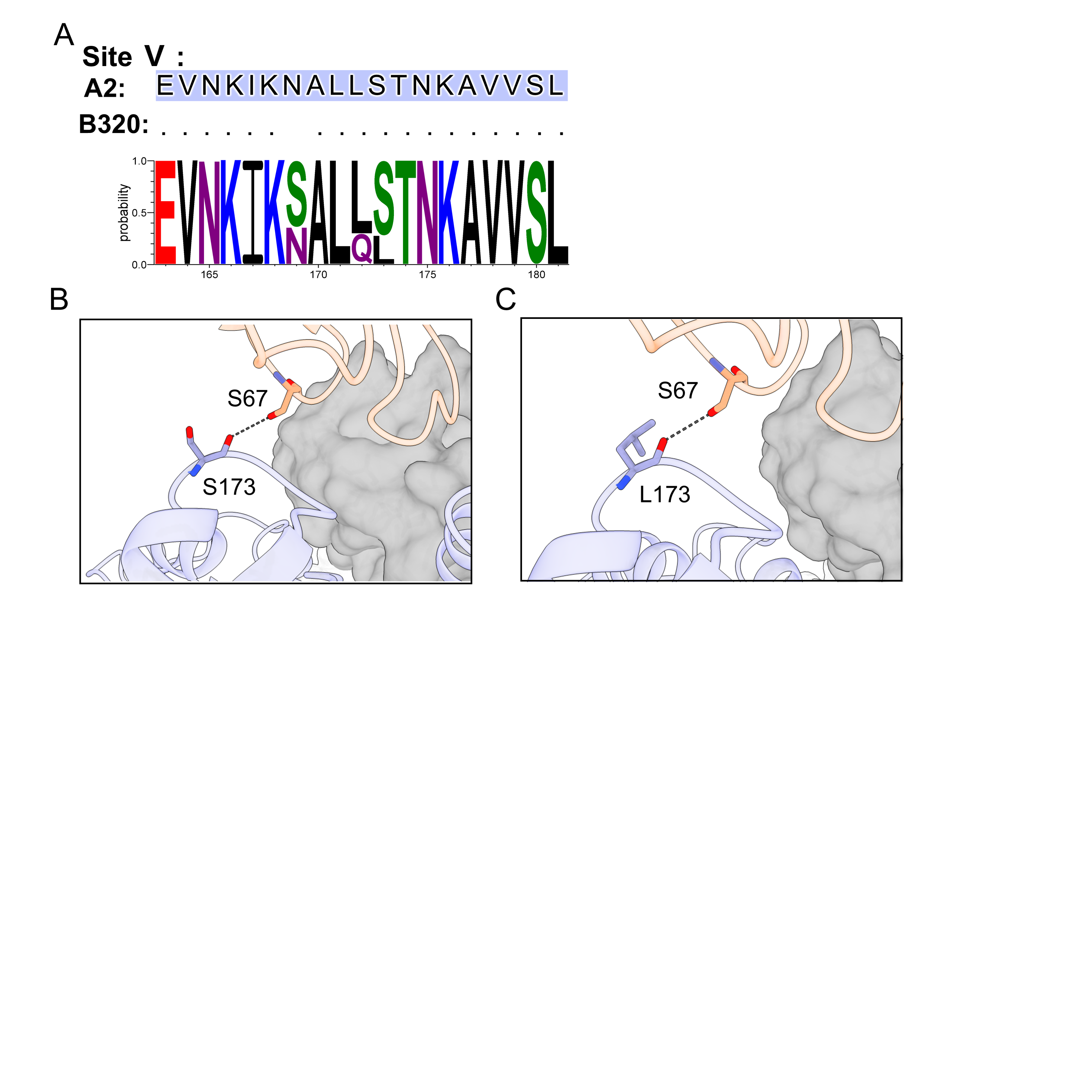

Supplement: S12 Fig — (A) Conservative analysis of site V. The sequence conservation of 2383 full-length RSV F genes in the last decade was downloaded from NCBI. Related to Fig 4B. (B) Close-up views of the interaction between S173 of RSV pre-F and S67 of the PR306007 VL domain. (C) Close-up views of the interaction between L173 mutation of RSV pre-F and S67 of the PR306007 VL domain. The VL of the PR306007 and a pre-F protomer are depicted as cartoons, in orange (light chain) and purple (pre-F), respectively. For clarity, key contact residues are labeled and displayed as sticks, with oxygen atoms in red and nitrogen atoms in blue. Hydrogen bonds are represented by black dashed lines. (TIF) [file ppat.1013674.s012.tif]

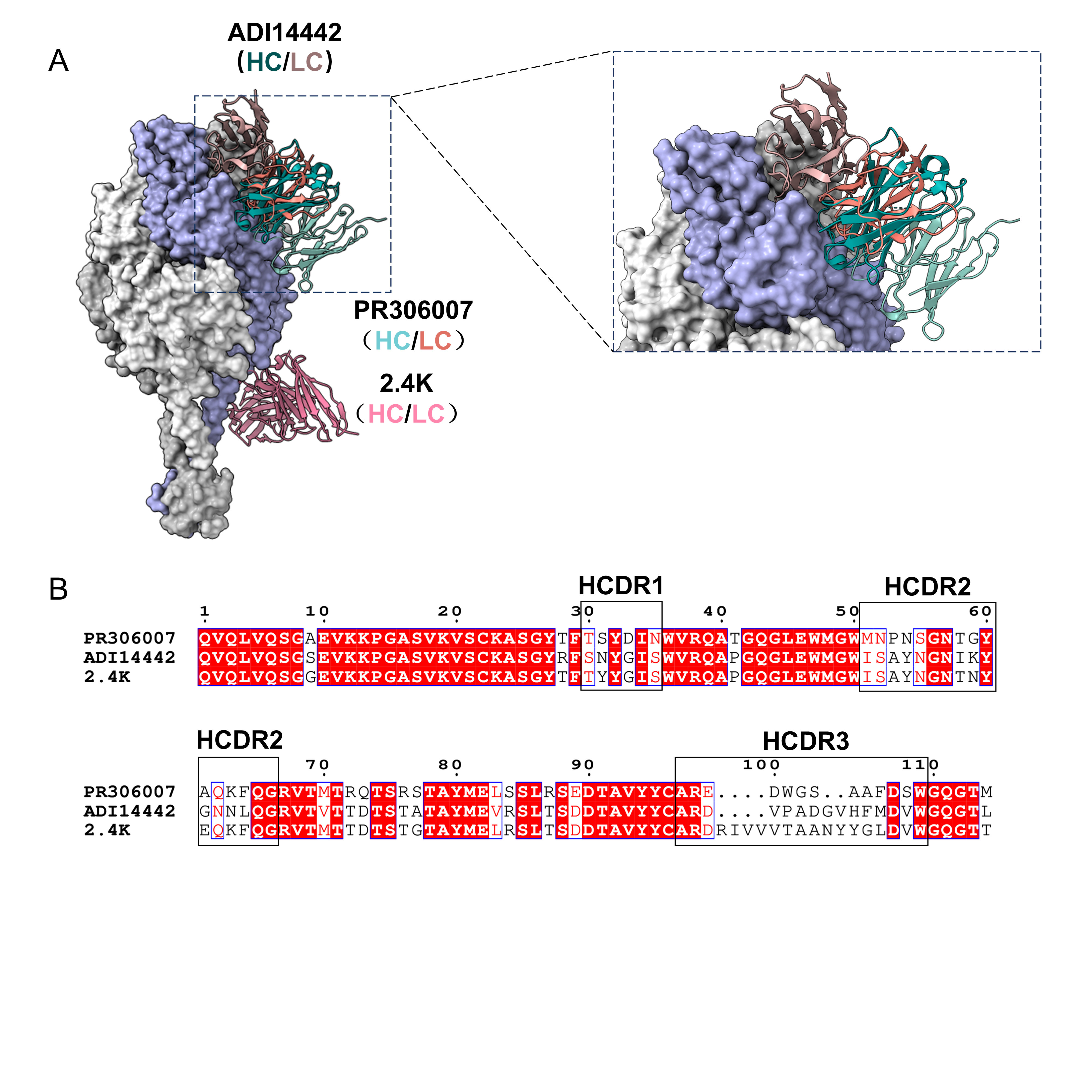

Supplement: S13 Fig — (A) Alignment of selected NAbs derived from VH1–18 germline. PR306007 (HC: peacock blue, LC: Dark), ADI14442 (HC: teal, LC: Brick red), and 2.4K (pink) are superimposed. RSV F is shown in the surface, a protomer colored purple and other two protomers colored grey. (B) Sequence alignment of the HC variable domain sequence of PR306007 to other reported antibodies from the VH1–18 germline. (TIF) [file ppat.1013674.s013.tif]
