# Supplementary material for: Discovery and molecular mechanism of potent neutralizing antibody from humanized mice with respiratory syncytial virus
Source: PLoS Pathog. 2025 Nov 17;21(11):e1013674. doi: 10.1371/journal.ppat.1013674 (PMC12637950; doi:10.1371/journal.ppat.1013674)
Supplement: S4 Table — (DOCX) [file ppat.1013674.s017.docx]

**Supplementary table 4**

**S4 Table. Cryo-EM data collection, and refinement.**

| **RSV pre-F-PR306007Fab** | | |
| --- | --- | --- |
| **Data Collection and processing** |  | |
| Magnification | | 22,500x |
| Voltage (keV) | | 300 |
| Electron exposure (e–/Å^2^) | | 60 |
| Defocus range (μm) | | 2.7 |
| Pixel size (Å) | | 1.07 |
| Number of movies | | 3225 |
| Initial particle images (no.) | | 2,462,209 |
| Final particle images (no.) | | 52,130 |
| Symmetry imposed | | C1 |
| Map resolution (Å) | | 4.08 |
| FSC threshold | | 0.143 |
| Map local resolution range (Å) | | 3.5-6.5 |
| **Refinement** | |  |
| Initial model used  (PDB code) | | 8F9U、4MMV |
| Model resolution (Å) | | 4.0 |
| FSC threshold | | 0.143 |
| Map sharpening *B* factor (Å^2^) | | -63 |
| **Model composition** | |  |
| Non-hydrogen atoms | | 12280 |
| Protein residues | | 1677 |
| Ligands (Phospholipid) | | 0 |
| **R.m.s. deviations** | |  |
| Bond lengths (Å) | | 0.003 |
| Bond angles (°) | | 0.621 |
| **Validation** | |  |
| MolProbity score | | 1.94 |
| Clashscore | | 9.21 |
| Rotamers outliers (%) | | 0 |
| **Ramachandran plot** | |  |
| Favored (%) | | 92.84 |
| Allowed (%) | | 6.98 |
| Outliers (%) | | 0.18 |
